# Supplementary material for: NET-02 trial protocol: a multicentre, randomised, parallel group, open-label, phase II, single-stage selection trial of liposomal irinotecan (nal-IRI) and 5-fluorouracil (5-FU)/folinic acid or docetaxel as second-line therapy in patients with progressive poorly differentiated extrapulmonary neuroendocrine carcinoma (NEC)
Source: BMJ Open. 2020 Feb 5;10(2):e034527. doi: 10.1136/bmjopen-2019-034527 (PMC7045240; doi:10.1136/bmjopen-2019-034527)
Supplement: Supplementary data [file bmjopen-2019-034527supp001.pdf]

Supplementary material

Contents

Supplementary material 1

1. TRIAL SITES 2
2. DOSE MODIFICATIONS 3

2.1 Liposomal irinotecan (nal-IRI) dose modifications 3

2.2 5-Fluorouracil (5-FU) and folinic acid dose modifications 5

2.2.1 Haematological toxicities: 5-FU dose modifications 5

2.2.2 Non-haematological toxicities: 5-FU dose modifications 5

2.2.3 Other toxicity requiring special attention 6

2.3 Docetaxel dose modifications 6

2.4 Management of infusion reactions 8

1. TRIAL SITES

Table 1 list the trial sites, which are taking part in the NET-02 trial, indicating if they are open to recruitment or are in set-up. The principal investigator (PI) of each site is listed and for sites that are open to recruitment, the number or patients randomised up to the point of submission are provided.

Table 1 | Trial sites

Site status	Site Name	PI Name	Number of patients randomised
Open	Christie Hospital	Dr Mairéad McNamara	8
	Weston Park Hospital	Dr Jon Wadsley	2
	Beatson	Professor Nick Reed	2
	Hammersmith Hospital	Dr Rohini Sharma	1
	Royal Free Hospital	Dr Daniel Krell	0
	Royal Marsden Hospital	Dr Ian Chau	1
	Western General Hospital	Dr Lucy Wall	1
	The Clatterbridge Cancer Centre	Dr Olusola Faluyi	1
	University Hospital Southampton	Dr Judith Cave	1
	Velindre Cancer Centre	Dr Carys Morgan	0
	Guy's Hospital	Dr Debashis Sarker	0
	Newcastle	Dr Jane Margetts	0
In set-up	University Hospital Coventry	Dr Sharmila Sothi	n/a
	Belfast	Dr Martin Eatock	n/a
	St James University Hospital	Dr Alan Anthoney	n/a
	Cheltenham General Hospital	Dr David Farrugia	n/a

2. DOSE MODIFICATIONS

2.1 Liposomal irinotecan (nal-IRI) dose modifications

For Grade 1 and 2 toxicities, no dose modifications are required. In the event of Grade 3 or 4 toxicity, the doses of liposomal irinotecan (nal-IRI) and 5-fluorouracil (5-FU) must be reduced and subsequent doses of nal-IRI and 5-FU must continue to be adjusted as indicated in Table 2. All dose modifications must be based on the worst preceding toxicity.

Table 2 | Dose modifications for nal-IRI and 5-FU and folinic acid, for Grade 3-4 toxicities.

	**Toxicity**<br>CTCAE Grade (value)	**Dose Adjustment**
**Haematological toxicities**	**Neutropenia**<br>Grade 3 or Grade 4<br>(<1000/mm³: <1x10⁹/L)<br>Or neutropenic fever	**A new cycle of therapy should not begin until the absolute neutrophil count is ≥1.5x10⁹/L (dose modifications below are for subsequent treatments, if grade 3 or 4 neutropenia is recorded on day 1 of a cycle or neutropenic fever is experienced during a cycle)***<br>**First occurrence** Reduce nal-IRI dose to 60 mg/m²<br>Reduce 5-FU dose by 25% (1800 mg/m²)<br>**Second occurrence** Reduce nal-IRI dose to 50 mg/m²<br>Reduce 5-FU dose by 25% (1350 mg/m²)<br>**Third occurrence** Discontinue treatment
	**Thrombocytopenia**<br>**Leukopenia**<br>Grade 3 or 4	**A new cycle of therapy should not begin until the platelet count is ≥100x10⁹/L**<br>Dose modifications for grade 3 or 4 thrombocytopenia are the same as recommended for neutropenia above for first, second and third recurrence.
**Non-haematological toxicities**	**Diarrhoea**<br>Grade 3 or 4<br>(≥7 stools per day pre-treatment)	**A new cycle of therapy should not begin until diarrhoea resolves to ≤Grade 1** (2-3 stools/day more than pre-treatment frequency) **(Dose modifications below are for subsequent treatments)**<br>**First occurrence** Reduce nal-IRI dose to 60 mg/m²<br>Reduce 5-FU dose by 25% (1800 mg/m²)<br>**Second occurrence** Reduce nal-IRI dose to 50 mg/m²<br>Reduce 5-FU dose by 25% (1350 mg/m²)

	Toxicity<br>CTCAE Grade (value)	Dose Adjustment
		**Third occurrence** Discontinue treatment
	**Nausea/vomiting**<br>Grade 3 or 4 despite optimal antiemetic therapy	**A new cycle of therapy should not begin until nausea/vomiting resolves to ≤Grade 1 or baseline (Dose modifications below are for subsequent treatments)**<br>**First occurrence** Optimise antiemetic therapy<br>Reduce nal-IRI dose to 60 mg/m^2<br>**Second occurrence** Optimise antiemetic therapy<br>Reduce nal-IRI dose to 50 mg/m^2<br>**Third occurrence** Discontinue treatment
	**Hepatic, renal, respiratory or other toxicities**<br>Grade 3 or 4<br>(asthenia and grade 3 anorexia do not require dose adjustment and also excluding grade ≥3 ALT/AST which resolve to baseline within 7 days and grade ≥3 toxicities which following case causality assessment are not in the category of 'Certain', 'Probable' or 'Possible' and as such are not related to study treatment, or which are not considered a clinically-significant toxicity)	**A new cycle of therapy should not begin until the adverse reaction resolves to ≤Grade 1 (Dose modifications below are for subsequent treatments)**<br>**First occurrence** Reduce nal-IRI dose to 60 mg/m^2<br>Reduce 5-FU dose by 25% (1800 mg/m^2)<br>**Second occurrence** Reduce nal-IRI dose to 50 mg/m^2<br>Reduce 5-FU dose by 25% (1350 mg/m^2)<br>**Third occurrence** Discontinue treatment

* Prophylactic use of granulocyte colony-stimulating factor (G-CSF) can be considered prior to dose modification in those patients who have had at least one episode of grade 3 or 4 neutropenia or neutropenic fever while receiving therapy or have had documented grade 3 or 4 neutropenia or neutropenic fever while receiving prior anti-neoplastic therapy.

CTCAE: Common Terminology Criteria for Adverse Events, ALT: alanine aminotransferase, AST: aspartate aminotransferase.

2.2 5-Fluorouracil (5-FU) and folinic acid dose modifications

Dose modifications for 5-FU are provided below. No dose adjustments for toxicity are required for folinic acid. Folinic acid must be given immediately prior to each 5-FU dose; hence, if the 5-FU dose is held, folinic acid dose should be held as well. If the dosing of nal-IRI needs to be withheld, then the 5-FU/folinic acid in the combination can be administered as monotherapy. In case a patient experiences an infusion reaction, either institutional guidelines or the guidelines provided for nal-IRI infusion reaction management (section 2.4) should be used.

2.2.1 Haematological toxicities: 5-FU dose modifications

Absolute neutrophil count (ANC) and platelet count should be measured locally no more than 3 days prior to day 1 of each treatment cycle. Treatment should only proceed if;

- ANC ≥ 1.5x10^9/L
- Platelet count ≥100x10^9/L

In the event of haematological toxicity, treatment should be delayed (up to 28 days) to allow sufficient time for recovery. On recovery, treatment should be administered according to the guidelines provided in Table 2.

2.2.2 Non-haematological toxicities: 5-FU dose modifications

Treatment should be delayed until all clinically significant Grade 3 or 4 non-haematological toxicities resolve to Grade 1 or baseline. If delays are greater than 28 days for toxicity, the participant should be withdrawn from trial treatment. Dose adjustments of other 5-FU-related toxicities are provided in Table 3. Asthenia, grade 3 anorexia and grade ≥3 toxicities which following case causality assessment are not in the category of 'Certain', 'Probable' or 'Possible' and as such are not related to study treatment, or which are not considered a clinically-significant toxicity, do not require dose modifications.

Table 3 | 5-FU dose modifications for other non-haematological toxicities

Worst toxicity CTCAE grade	5-FU dose for next cycle[a]
Grade 1 or 2	100% of previous dose, except for Grade 2 hand-foot syndrome, Grade 2 cardiac toxicity, or any grade neurocerebellar toxicity
Grade 2 hand-foot syndrome	Reduce dose by 25%[b]
Any grade neurocerebellar or ≥ Grade 2 cardiac toxicity	Discontinue therapy
Grade 3 or 4	Reduce dose by 25%[b], except for Grade 3 or 4 hand-foot syndrome
Grade 4 or 4 hand-foot syndrome	Discontinue therapy

[a] All dose modifications must be based on the worst preceding toxicity.

[b] Participants who require more than 2 dose reductions should be withdrawn from trial treatment unless agreed with the Chief Investigator or delegate.

CTCAE: Common Terminology Criteria for Adverse Events, 5-FU; 5-Fluorouracil.

2.2.3 Other toxicity requiring special attention

Corrected QT interval (QTc) prolongation that occurs in the setting of diarrhoea-induced electrolyte imbalance should be treated with appropriate electrolyte repletion. Once the underlying abnormality is corrected and the electrocardiogram (ECG) abnormalities have reversed, treatment may continue under careful monitoring, and with appropriate dose modification for diarrhoea as per local standard of care practice.

2.3 Docetaxel dose modifications

Neutrophil and platelet count should be measured locally no more than 3 days prior to day 1 of each treatment cycle. Treatment should only proceed if:

- ANC ≥ $1.5x10^9$/L
- Platelet count ≥$100x10^9$/L
- Bilirubin ≤1.5 x upper limit of normal (ULN)
- Alanine aminotransferase (ALT) and/or Aspartate aminotransferase (AST) ≤2.5 x ULN (in absence of liver metastasis) or ≤5 x ULN (in presence of liver metastasis)

In the event of haematological toxicity, treatment should be delayed (up to 28 days) to allow sufficient time for recovery. Guidelines for docetaxel dose modifications are provided in Table 4.

Table 4 | Dose reductions for docetaxel toxicities

Toxicity	Severity	Management
**Hypersensitivity**	Grade 3/Grade 4	Administration of appropriate medication (see below).
**Neutropenia**	Day 1 neutrophil count <1500/mm^3: <1.5x10^9/L	Stop treatment until neutrophils recovers to at least 1.5x10^9/L. If neutrophils <1.5x10^9/L for ≤7 days, restart docetaxel at full dose (75mg/m^2). If neutrophils <1.5x10^9/L for >7 days, restart docetaxel at 55mg/m^2 or next lowest dose level (40mg/m^2) if already reduced.*
	Febrile neutropenia OR prolonged Grade 4 neutropenia (Neutrophil count <500/mm^3: <0.5x10^9/L for 7 days or more)	Stop treatment until neutrophils ≥ 1.5x10^9/L. Restart drug at 55mg/m^2 or next lowest dose level (40mg/m^2) if already reduced.*
**Neuropathy**	Grade 3/Grade 4	Stop docetaxel treatment.
**Thrombocytopenia**	Platelet count <100 x 10^9/L	Stop treatment until platelets ≥100x^9/L. Restart drug at full dose (75mg/m^2).
	Platelet count <50 x 10^9/L (Grade 3/Grade 4)	Stop treatment until platelets ≥100x^9/L. Restart drug at 55mg/m^2 or next lowest dose level (40mg/m^2) if already reduced.
**Hepatic Dysfunction**	Bilirubin >1.5 ULN<br>ALT/AST>2.5 x ULN (in absence of liver metastasis), >5 x ULN (in presence of liver metastasis)	Stop docetaxel until parameters recover to baseline levels. Restart drug at 55mg/m^2 or next lowest dose level (40mg/m^2) if already reduced.
**Cutaneous reaction**	Grade 2	Stop treatment until recovery to Grade 1 or better. Restart drug at full dose (75mg/m^2).
	Severe or cumulative<br>(Grade 3/Grade 4)	Stop treatment until recovery (Grade 1 or better). Restart drug at 55 mg/m^2 or next

Toxicity	Severity	Management
		lowest dose level (40mg/m2) if already reduced.
**Other non-haematological toxicity (excluding grade ≥3 toxicities which following case causality assessment are not in the category of 'Certain', 'Probable' or 'Possible' and as such is not related to study treatment, or which are not considered a clinically-significant toxicity)**	Grade 3/Grade 4	Stop docetaxel until parameters recover to baseline levels. Restart drug at 55mg/m^2 or next lowest dose level (40mg/m^2) if already reduced.

* Prophylactic use of granulocyte colony-stimulating factor (G-CSF) can be considered prior to dose modification in those patients who have had at least one episode of grade 3 or 4 neutropenia or neutropenic fever while receiving therapy or have had documented grade 3 or 4 neutropenia or neutropenic fever while receiving prior anti-neoplastic therapy.
ULN: upper limit of normal, ALT: alanine aminotransferase, AST: aspartate aminotransferase.

2.4 Management of infusion reactions

The guidelines described in this section can be followed in case of infusion reactions. Infusion reactions will be defined according to the National Cancer Institute Common Terminology Criteria for Adverse Events (CTCAE) (Version 5.0) definitions of an allergic reaction or anaphylaxis. Institutional policies or the following treatment guidelines shall be used for the management of infusion reactions.

Grade 1

- Slow infusion rate by 50%.
- Monitor patient every 15 minutes for worsening of condition.
- Future infusions may be administered at a reduced rate (e.g. over 120 minutes for nal-IRI), at the discretion of the Investigator.

Grade 2

- Stop infusion.

- Administer diphenhydramine hydrochloride 50 mg intravenously (IV) (or similar), acetaminophen 650 mg (or similar) orally, and oxygen.
- Resume infusion at 50% of the prior rate once infusion reaction has resolved.
- Monitor patient every 15 minutes for worsening of condition.
- For all subsequent infusions, pre-medicate with diphenhydramine hydrochloride 50 mg IV (or similar), dexamethasone 10 mg IV, and acetaminophen 650 mg (or similar) orally.
- Future infusions may be administered at a reduced rate (e.g. over 120 minutes for nal-IRI), at the discretion of the Investigator.

Grade 3

- Stop infusion and disconnect infusion tubing from patient.
- Administer diphenhydramine hydrochloride 50 mg IV (or similar), dexamethasone 10 mg IV, bronchodilators for bronchospasm, and other medications or oxygen as medically necessary.
- No further treatment will be permitted.

Grade 4

- Stop the infusion and disconnect infusion tubing from patient.
- Administer epinephrine (adrenaline), bronchodilators or oxygen as indicated for bronchospasm.
- Administer diphenhydramine hydrochloride 50 mg IV (or similar), dexamethasone 10 mg IV and other medications as medically necessary.
- Consider hospital admission for observation.
- No further treatment will be permitted.

For patients who experience a Grade 1 or Grade 2 infusion reaction, future infusions may be administered at a reduced rate (over 120 minutes), at the discretion of the treating physician. For patients who experience a second grade 1 or 2 infusion reaction, administer dexamethasone 10 mg IV. All subsequent infusions should be pre-medicated with diphenhydramine hydrochloride 50 mg IV (or similar), dexamethasone 10 mg IV, and acetaminophen 650 mg orally (or similar).
