# Supplementary material for: NET-02 trial protocol: a multicentre, randomised, parallel group, open-label, phase II, single-stage selection trial of liposomal irinotecan (nal-IRI) and 5-fluorouracil (5-FU)/folinic acid or docetaxel as second-line therapy in patients with progressive poorly differentiated extrapulmonary neuroendocrine carcinoma (NEC)
Source: BMJ Open. 2020 Feb 5;10(2):e034527. doi: 10.1136/bmjopen-2019-034527 (PMC7045240; doi:10.1136/bmjopen-2019-034527)
Supplement: Author's manuscript [file bmjopen-2019-034527.draft_revisions.pdf]

BMJ Open

BMJ Open is committed to open peer review. As part of this commitment we make the peer review history of every article we publish publicly available.

When an article is published we post the peer reviewers' comments and the authors' responses online. We also post the versions of the paper that were used during peer review. These are the versions that the peer review comments apply to.

The versions of the paper that follow are the versions that were submitted during the peer review process. They are not the versions of record or the final published versions. They should not be cited or distributed as the published version of this manuscript.

BMJ Open is an open access journal and the full, final, typeset and author-corrected version of record of the manuscript is available on our site with no access controls, subscription charges or pay-per-view fees (http://bmjopen.bmj.com).

If you have any questions on BMJ Open's open peer review process please email info.bmjopen@bmj.com

BMJ Open

The NET-02 trial protocol: A multi-centre, randomised, parallel group, open-label, phase II, single-stage selection trial of liposomal irinotecan (nal-IRI) and 5-fluorouracil (5-FU)/folinic acid or docetaxel as second-line therapy in patients with progressive poorly differentiated extra-pulmonary neuroendocrine carcinoma (NEC)

Journal:	*BMJ Open*
Manuscript ID	bmjopen-2019-034527
Article Type:	Protocol
Date Submitted by the Author:	24-Sep-2019
Complete List of Authors:	Craig, Zoe; University of Leeds, Clinical Trials Research Unit, Leeds Institute of Clinical Trials Research<br>Swain, Jayne; University of Leeds, Clinical Trials Research Unit, Leeds Institute of Clinical Trials Research<br>Batman, Emma; University of Leeds, Clinical Trials Research Unit, Leeds Institute of Clinical Trials Research<br>Wadsley, Jonathan; Weston Park Hospital<br>Reed, Nicholas; Beatson West of Scotland Cancer Centre<br>Faluyi, Olusola; Clatterbridge Cancer Centre NHS Foundation Trust<br>Cave, Judith; University Hospital Southampton NHS Foundation Trust, Department of Oncology<br>Sharma, Rohini; Imperial College London<br>Chau, Ian; Royal Marsden Hospital NHS Trust<br>Wall, Lucy; Western General Hospital<br>Lamarca, Angela; The Christie NHS Foundation Trust, Department of Medical Oncology; The University of Manchester, Division of Cancer Sciences<br>Hubner, R; The Christie NHS Foundation Trust, Department of Medical Oncology; The University of Manchester, Division of Cancer Sciences<br>Mansoor, Wasat; The Christie NHS Foundation Trust, Department of Medical Oncology<br>Sarker, Debashis; King's College Hospital<br>Meyer, Tim; University College London Cancer Institute<br>Cairns, David; University of Leeds, Clinical Trials Research Unit, Leeds Institute of Clinical Trials Research<br>Howard, Helen; University of Leeds, Clinical Trials Research Unit, Leeds Institute of Clinical Trials Research<br>Valle, Juan; The Christie NHS Foundation Trust, Department of Medical Oncology; The University of Manchester, Division of Cancer Sciences<br>McNamara, Mairéad ; The Christie NHS Foundation Trust, Department of Medical Oncology; The University of Manchester, Division of Cancer Sciences
Keywords:	Neuroendocrine carcinoma, Randomised, Single-stage, Liposomal irinotecan, Docetaxel

SCHOLARONE™
Manuscripts

I, the Submitting Author has the right to grant and does grant on behalf of all authors of the Work (as defined in the below author licence), an exclusive licence and/or a non-exclusive licence for contributions from authors who are: i) UK Crown employees; ii) where BMJ has agreed a CC-BY licence shall apply, and/or iii) in accordance with the terms applicable for US Federal Government officers or employees acting as part of their official duties; on a worldwide, perpetual, irrevocable, royalty-free basis to BMJ Publishing Group Ltd ("BMJ") its licensees and where the relevant Journal is co-owned by BMJ to the co-owners of the Journal, to publish the Work in this journal and any other BMJ products and to exploit all rights, as set out in our licence.

The Submitting Author accepts and understands that any supply made under these terms is made by BMJ to the Submitting Author unless you are acting as an employee on behalf of your employer or a postgraduate student of an affiliated institution which is paying any applicable article publishing charge ("APC") for Open Access articles. Where the Submitting Author wishes to make the Work available on an Open Access basis (and intends to pay the relevant APC), the terms of reuse of such Open Access shall be governed by a Creative Commons licence – details of these licences and which Creative Commons licence will apply to this Work are set out in our licence referred to above.

Other than as permitted in any relevant BMJ Author's Self Archiving Policies, I confirm this Work has not been accepted for publication elsewhere, is not being considered for publication elsewhere and does not duplicate material already published. I confirm all authors consent to publication of this Work and authorise the granting of this licence.

For peer review only

The NET-02 trial protocol: A multi-centre, randomised, parallel group, open-label, phase II, single-stage selection trial of liposomal irinotecan (nal-IRI) and 5-fluorouracil (5-FU)/folinic acid or docetaxel as second-line therapy in patients with progressive poorly differentiated extra-pulmonary neuroendocrine carcinoma (NEC)

Authors: Craig Zoe[1], Swain Jayne[1], Batman Emma[1], Wadsley Jonathan[2], Reed Nicholas[3], Faluyi Olusola[4], Cave Judith[5], Sharma Rohini[6], Chau Ian[7], Wall Lucy[8], Lamarca Angela[9,10], Hubner Richard A[9,10], Mansoor Wasat[9], Sarker Debashis[11], Meyer Tim[12], Cairns David A[1], Howard Helen[1], Valle Juan W[9,10], McNamara Mairéad G[9,10*]

[1]*Clinical Trials Research Unit, Leeds Institute of Clinical Trials Research, University of Leeds, Leeds, United Kingdom*

[2]*Weston Park Hospital, Sheffield, United Kingdom*

[3]*Beatson Oncology Centre, Glasgow, United Kingdom*

[4]*Clatterbridge Cancer Centre, Wirral, United Kingdom*

[5]*University Hospital Southampton, Southampton, United Kingdom*

[6]*Imperial College, London, United Kingdom*

[7]*Royal Marsden, London, United Kingdom*

[8]*Western General Hospital, Edinburgh, United Kingdom*

[9]*Department of Medical Oncology, The Christie NHS Foundation Trust, Manchester, United Kingdom*

[10]*Division of Cancer Sciences, University of Manchester, Manchester, United Kingdom*

[11]*King's College Hospital, London, United Kingdom*

[12]*University College London Cancer Institute, London*, United Kingdom

* *Corresponding author:* Mairead.McNamara@christie.nhs.uk

Department of Medical Oncology,

The Christie NHS Foundation Trust/University of Manchester,

Wilmslow Road,

Withington,

Manchester M20 4BX,

United Kingdom

0161 446 8106

Word count: 3,991

For peer review only

ABSTRACT

Introduction: Poorly differentiated (PD), extra-pulmonary (EP), neuroendocrine carcinomas (NECs) are rare but aggressive neuroendocrine neoplasms (NENs). First-line treatment for advanced disease is an etoposide-platinum-based chemotherapy combination. There is no established second-line treatment for patients with PD-EP-NEC, and this is an area of unmet need.

Methods and analysis: NET-02 is a UK, multi-centre, randomised (1:1), parallel group, open-label, phase II, single-stage selection trial of liposomal irinotecan (nal-IRI)/5-fluorouracil (5-FU)/folinic acid or docetaxel as second-line therapy in patients with progressive PD-EP-NEC. One hundred and two eligible participants will be randomised to receive either nal-IRI/5-FU/folinic acid or docetaxel. The primary objective is to determine the 6-month progression-free survival (PFS) rate. The secondary objectives of this study are to determine PFS, overall survival, objective response rate, toxicity, quality of life and whether neuron-specific enolase is predictive of treatment response. If either treatment is found to have a 6-month PFS rate of at least 25%, that treatment will be considered for a phase III trial. If both treatments meet this target, pre-specified selection criteria will be applied to establish which treatment to take forward.

Ethics and dissemination: This study has ethical approval from the Greater Manchester Central Research Ethics Committee (Reference No. 18/NW/0031) and clinical trial authorisation from the Medicine and Healthcare Products Regulatory Agency. Results will be published in peer-reviewed journals and uploaded to the EU Clinical Trials Register.

Trial registration: ISRCTN10996604, ClinicalTrials.gov: NCT03837977, EudraCT Number: 2017-002453-11

Key words: Neuroendocrine carcinoma, randomised, single-stage, liposomal irinotecan, docetaxel

For peer review only

ARTICLE SUMMARY

Strengths and limitations of this study

- The trial is designed to ensure, with a high probability, that the most efficacious treatment is selected to be taken forward to a phase III trial.
- Prospectively defined decision criteria in this trial will enable earlier planning of a phase III trial if these targets are reached.
- The trial is not powered to directly compare the two treatment arms in this study.

For peer review only

For peer review only - http://bmjopen.bmj.com/site/about/guidelines.xhtml

INTRODUCTION

Neuroendocrine carcinomas

Neuroendocrine carcinomas (NECs) are a rare, high-grade, poorly differentiated (PD) form of neuroendocrine neoplasms (NENs).[1] The annual incidence of PD extra-pulmonary (EP) NEC is approximately one diagnosis per 100,000 persons.[2 3] These tumours are characterised by aggressive histological features; high Ki-67 index (>20% by definition, but usually higher (>75%)),[4] extensive necrosis and nuclear atypia, and are classified as NEC grade 3 according to the World Health Organisation (WHO) 2010 classification.[5]

First-line treatment for PD-EP-NECs has remained largely unchanged since a study in the early 1990s reported anti-tumour activity and high tumour response rates (RRs) produced by an etoposide-platinum combination.[6] Nevertheless, disease progression invariably occurs in patients during or following completion of first-line therapy, and a standard second-line treatment is yet to be determined.

Current second-line treatment options for patients with a NEC diagnosis

For patients with advanced PD-EP-NEC, combination regimens such as irinotecan, 5-fluorouracil (5-FU) and folinic acid are a second-line treatment option currently used, without robust trial evidence.[7] This combination has been recommended for patients with a NEC diagnosis with a Ki-67 ≥55%, whereas some literature recommends temozolomide based-combinations for those with a Ki-67 <55%.[8 9] In devising treatment strategies for PD-EP-NEC, many refer to the extensive literature on high-grade NEC of the lung, for which docetaxel is a second-line therapy option.[9]

Several small retrospective studies have published results for the outcomes of second-line chemotherapy after failure of the etoposide-platinum combination in patients with grade 3 NECs.[7-13] The NORDIC-NEC study reported predictive and prognostic factors for treatment and survival in 305

For peer review only - http://bmjopen.bmj.com/site/about/guidelines.xhtml

patients with advanced gastrointestinal NEC.[9] Second-line chemotherapy was administered to 100 patients; of these, 35 received temozolomide-based chemotherapy and 20 received docetaxel-based chemotherapy. Of 84 evaluable patients, the RR was 18%. Those whose tumours had a Ki-67 <55% had a lower RR, but better survival than patients whose tumours had a Ki-67 ≥55%. The median overall survival (OS) for patients treated with first-line platinum-based chemotherapy in the advanced setting is 11-16.4 months.[9 14] In a systematic review and meta-analysis of second-line treatment in 595 patients with advanced PD-EP-NEC, the median RR was 18%, the median progression-free survival (PFS) was 2.5 months (range 1.2-6.0) and the median OS was 7.6 months (range 3.2-22).[15]

Liposomal irinotecan (nal-IRI)

Irinotecan, a topoisomerase I inhibitor, works to arrest uncontrolled cell growth by preventing the unwinding of deoxyribonucleic acid (DNA), therefore preventing cell replication and tumour growth.[16]

Liposomal irinotecan (nal-IRI) (ONIVYDE®, Servier) is irinotecan encapsulated in a liposome drug delivery system. This stable liposome formulation of irinotecan has several attributes that may provide an improved therapeutic index; controlled and sustained release, high intravascular drug retention and enhanced permeability.[16 17] The improved pharmacokinetics and bio-distribution of nal-IRI in comparison to irinotecan may have clinical benefit in patients with NEC.

Pharmacokinetic studies have demonstrated that once irinotecan is released from the liposomes, the conversion to the active metabolite, SN-38, is similar to that of un-encapsulated irinotecan.[16 18] Thus, nal-IRI and un-encapsulated irinotecan have demonstrated similar adverse reactions (ARs) in patients, the most common of which include gastrointestinal events and myelosuppression.[16 18]

For peer review only - http://bmjopen.bmj.com/site/about/guidelines.xhtml

Rationale for the use of nal-IRI in combination with 5-FU and folinic acid

Pre-clinical evidence supports the hypothesis that nal-IRI modifies the tumour microenvironment in a manner that should make tumours more susceptible to 5-FU/folinic acid, through decreasing tumour hypoxia and increasing small molecule perfusion.[19 20]

Given the relative absence of overlapping toxic effects among nal-IRI, 5-FU and folinic acid, a regimen combining these agents was studied in a phase I, dose-escalation trial of solid tumours.[21] Among the 15 efficacy-evaluable participants, the overall disease control rate was 73.3%. Among the six participants who received the nal-IRI maximum tolerated dose of 80 mg/m^2, the objective response rate (ORR) and disease control rate were 16.7% and 83.3%, respectively.

In the NAPOLI-1 phase III trial of nal-IRI, with or without 5-FU and folinic acid, versus 5-FU and folinic acid alone, in the treatment of patients with metastatic pancreatic ductal adenocarcinoma after receiving gemcitabine-based therapy, an increase in OS for those treated with a combination of nal-IRI and 5-FU/folinic acid was reported compared to those treated with 5-FU and folinic acid alone (hazard ratio for survival (HR) 0.67, 95% confidence interval (CI) 0.49-0.92).[22]

Rationale for the use of docetaxel

The National Comprehensive Cancer Network Clinical Practice Guidelines in Oncology (NCCN Guidelines®), for the treatment of small cell and non-small cell lung cancer, include docetaxel as a second-line treatment option in patients who have progressed on primary etoposide-platinum combination therapy.[23 24] Based on observed RRs, survival, quality of life (QoL) and toxicities, the optimal dose of docetaxel in pre-treated patients with non-small cell lung cancer is 75mg/m^2 every 3 weeks.[25]

For peer review only

Study rationale and aim

Treatment of patients with advanced PD-EP-NEC, to date, has been analogous to that of high-grade NEC (small cell or non-small cell cancer) of the lung.[6] The standard arm of NET-02 is that used in high-grade lung NEC, of which docetaxel is a second-line therapy option,[23] and combination regimens such as irinotecan/5-FU are a second-line therapy option currently used, without trial evidence, for this subset of patients.[7] Prospective collaborative trials, with translational end-points, are warranted and may inform future biomarker-driven studies.

Therefore, the overall aim of this trial is to assess the efficacy of nal-IRI/5-FU/folinic acid or docetaxel, separately, as second-line therapy in patients with progressive PD-EP-NEC, with selection criteria applied to establish which treatment to take forward into a phase III trial.

METHODS AND ANALYSIS

Trial objectives

The primary objective of the trial is to determine the 6-month PFS rate, defined as a binary outcome (progression-free or not) within the timeframe of treatment start date until 6 months post-randomisation.

The secondary objectives of the trial are to determine:

- Progression-free survival (defined as the time from randomisation to progression or death from any cause)
- Overall survival (defined as the time from randomisation to death from any cause)
- Objective response rate at 6 months post-randomisation (defined using the Response Evaluation Criteria in Solid Tumours (RECIST) v1.1 measurements)[26]
- Toxicity as per Common Terminology Criteria for Adverse Events (CTCAE) version 5.0
- Quality of life (defined using European Organisation for Research and Treatment of Cancer

For peer review only - http://bmjopen.bmj.com/site/about/guidelines.xhtml

(EORTC) quality of life validated questionnaires (QLQ) C30 (EORTC QLQ-C30)[27] and GINET21 (EORTC QLQ-GINET21))[27 28]

- Association between neuron-specific enolase concentration and treatment response

Additional exploratory objectives, analysing participant samples, will include:

- Quantification of circulating tumour cells (CTCs) and circulating tumour DNA at baseline, 6 weeks and on progression, to identify any correlation with disease-related outcomes
- Molecular profiling of CTCs, circulating tumour DNA and tumour tissue (further immunohistochemistry on tumour tissue may also be required) to identify any correlation with disease-related outcomes
- Generation of mouse models of PD-EP-NEC

Trial design

The NET-02 trial is a United Kingdom (UK), multi-centre, randomised (1:1), parallel group, open-label, phase II, single-stage selection trial of nal-IRI/5-FU/folinic acid or docetaxel as second-line therapy in patients with progressive PD-EP-NEC.

The design is an adaptation of a one-stage trial design proposed by Simon, Wittes and Ellenberg, where the A'Hern design is first implemented to assess efficacy of each treatment separately, to ensure a pre-specified minimum level of activity prior to selection.[29] Should both treatments be sufficiently efficacious, pre-specified selection criteria are then applied to establish which treatment to take forward into a phase III trial. The intention of the trial is to show that the regimens are sufficiently active in this patient population, but not to show that one regimen is significantly superior to the other.

For peer review only - http://bmjopen.bmj.com/site/about/guidelines.xhtml

The A'Hern method is advantageous over other single-stage designs, since it uses the exact binomial distribution, as opposed to a normal approximation to the binomial distribution which can lead to substantial error in small trials.[30] Additionally, prospectively defined decision criteria, specified below, are applied, which if reached, could enable earlier planning for a phase III follow-on trial.

Participants will be randomised to receive either nal-IRI/5-FU/folinic acid, administered every 14 days, or docetaxel, administered every 21 days. Trial treatment will continue until progressive disease, intolerable toxicity, delay of treatment for more than 28 days, development of any condition or occurrence of any event, which, in the opinion of the local investigator, justifies discontinuation of treatment, participant request or until 6 months after the last participant is randomised, whichever occurs first. Figure 1 displays the full trial schema.

Trial population and sample size

The NET-02 trial will recruit patients diagnosed with PD-EP-NEC (Ki-67 >20% and grade 3, confirmed by histology). Patients will be eligible for the trial if they meet all of the inclusion criteria and do not satisfy any of the exclusion criteria listed in Table 1.

Table 1 | NET-02 inclusion and exclusion criteria

Inclusion criteria	Exclusion criteria
1. Age ≥18 years and life expectancy >3 months.<br>2. Diagnosed with poorly differentiated (as defined by the WHO in 2010, Ki-67 >20%) EP-NEC (grade 3, confirmed by histology). Carcinoma of unknown primary is allowed if lung primary has been excluded following review by the multi-disciplinary team.<br>3. Prior treatment with first-line platinum-based chemotherapy for NEC in the advanced setting and ≥28 days from Day 1 of the previous treatment cycle.<br>4. Documented radiological evidence of disease progression OR discontinuation of first-line platinum-based chemotherapy due to intolerance.<br>5. Measurable disease according to RECIST 1.1.<br>6. Eastern Co-operative Oncology Group (ECOG) performance status ≤2.	1. Known or suspected allergy or hypersensitivity reaction to any of the components of study treatment or their excipients.<br>2. Use (including self-medication) within one week of randomisation and for the duration of the study of any of the following: St. John's wort, grapefruit, Seville oranges, medicines known to inhibit UGT1A1 (e.g. atazanavir, gemfibrozil, indinavir) and medicines known to inhibit or induce either CYP3A4 or CYP3A5.<br>3. Previous treatment (for NEC) with any of the components of combination chemotherapy regimens detailed in this study (nal-IRI, 5-FU, irinotecan, topoisomerase inhibitors or taxane-based therapy).<br>4. Incomplete recovery from previous therapy in the opinion of the investigator (surgery/adjuvant therapy/radiotherapy/chemotherapy in

For peer review only - http://bmjopen.bmj.com/site/about/guidelines.xhtml

Inclusion criteria	Exclusion criteria
7. Adequate renal function with serum creatinine ≤1.5 times upper limit of normal (ULN) and creatinine clearance ≥30ml/min according to Cockroft-Gault or Wright formula. If the calculated creatinine clearance is <30ml/min, glomerular filtration rate (GFR) may be assessed using either Cr51-EDTA or 99mTc-DTPA clearance method to confirm if GFR is ≥30 ml/min).<br>8. Adequate haematological function: Hb ≥90g/L, WBC ≥3.0 x 10^9/L, ANC ≥1.5 x 10^9/L, platelet count ≥100 x 10^9/L.<br>9. Adequate liver function: serum total bilirubin ≤1.5 x ULN (biliary drainage is allowed for biliary obstruction) and ALT and/or AST ≤2.5 x ULN in the absence of liver metastases, or ≤5 x ULN in the presence of liver metastases.<br>10. A negative pregnancy test is required at registration in women of childbearing potential.<br>11. Men and women of reproductive potential must agree to use a highly effective form of contraception during the study and for 6 months following the last dose of trial treatment. In addition, male participants should use a condom during study participation and for 6 months following the last dose of trial treatment.<br>12. Patients must be able to provide written informed consent.<br>13. Patients must be able and willing to comply with the terms of the protocol.	advanced setting), including ongoing peripheral neuropathy of > Common Terminology Criteria for Adverse Events (CTCAE) v5.0 grade 2 from previous platinum-based therapy.<br>5. Concurrent palliative radiotherapy involving target lesions used for this study (<28 days from discontinuation of radiotherapy). Radiotherapy for non-target lesions is allowed if other target lesions are available outside the involved field.<br>6. Patients must not have a history of other malignant diseases (within the previous 3 years, and there must be no evidence of recurrence), other than:<br>• EP-NEC.<br>• Non-melanoma skin cancer where treatment consisted of resection only or radiotherapy.<br>• Ductal carcinoma *in situ* (DCIS) where treatment consisted of resection only.<br>• Cervical carcinoma *in situ* where treatment consisted of resection only.<br>• Superficial bladder carcinoma where treatment consisted of resection only.<br>7. Documented brain metastases, unless adequately treated (surgery or radiotherapy only), with no evidence of progression and neurologically stable off anticonvulsants and steroids.<br>8. Clinically significant gastrointestinal disorder (in the opinion of the treating clinician), including hepatic disorders, bleeding, inflammation, obstruction, or diarrhoea > CTCAE grade 1 (at time of study entry).<br>9. Severe arterial thromboembolic events (myocardial infarction, unstable angina pectoris, stroke) less than 6 months before inclusion.<br>10. New York Heart Association (NYHA) Class III or IV congestive heart failure, ventricular arrhythmias or uncontrolled blood pressure.<br>11. Severe bone marrow failure or bone marrow depression after radiotherapy or treatment with other antineoplastic agents (defined as haematological values of haemoglobin or white blood cells or neutrophils or platelets not meeting inclusion criteria).<br>12. Known active hepatitis B virus, hepatitis C virus or HIV infection.<br>13. Active chronic inflammatory bowel disease.<br>14. Breastfeeding women.<br>15. Evidence of severe or uncontrolled systemic diseases, which, in the view of the treating clinician, makes it undesirable for the patient to participate in the trial.<br>16. Evidence of significant clinical disorder or laboratory finding which, in the opinion of the

For peer review only

Inclusion criteria	Exclusion criteria
	treating clinician, makes it undesirable for the patient to participate in the trial.<br>17. Medical or psychiatric conditions that impair the ability to give informed consent.<br>18. Any other serious uncontrolled medical conditions (in the opinion of the treating clinician).

One hundred and two eligible participants will be randomised to receive either nal-IRI/5-FU/folinic acid or docetaxel. Allowing for a 5% drop-out rate, this will provide 80% power for demonstrating that the one-sided 95% CI for the 6-month PFS rate excludes 15%, if the true rate is at least 30%, where 30% is the required level of efficacy and a rate of 15% or less would give grounds for rejection, i.e. the relevant treatment would be considered not to have reached an acceptable level of efficacy to warrant further evaluation. The proportions of 15% and 30% were chosen in line with existing literature; of those who reported the proportion progression-free at 6 months, the lowest was approximately 15%[11] and the highest approximately 25%.[7] Therefore, for either trial treatment to be taken forward for further research, they should provide estimates that are at least as good as the lower value and aim to improve on the higher value.

A treatment arm may be considered for further evaluation using the treatment selection process described below, if at least 12 out of 48 evaluable participants are progression-free at 6 months (equating to a success rate of 25%, with a lower one-sided 95% confidence limit of 15.1%).

Treatment selection

If both treatments successfully exceed the pre-defined criteria, having lower one-sided 95% confidence limits greater than 15%, Simon's design proposes that the treatment with the higher PFS rate at 6 months should be selected, regardless how small its advantage over the other treatment appears.[29] Nevertheless, to ensure that the more efficacious treatment is selected with a high probability, if the difference in the 6-month PFS rates is less than 5%, additional selection criteria,

For peer review only - http://bmjopen.bmj.com/site/about/guidelines.xhtml

including toxicity rates and QoL score, will be considered. If only one of the treatments successfully exceeds the pre-defined criteria, this treatment will be selected for further investigation.

Recruitment, registration and randomisation

Participants will be recruited from 16 UK sites (see supplementary material) over a 37-month period. Potential participants will be approached, regarding trial participation, during the standard clinic visit at which their progression following first-line chemotherapy is discussed and will be provided with a verbal and written explanation of the trial. Patients, who provide written informed consent, to the site Principal Investigator or delegate, will be registered onto the trial. Consent to the use of blood samples for future projects and mouse model generation (The Christie NHS Foundation Trust participants only) is optional.

Recruitment of participants to the NET-02 trial requires trial-specific investigations to confirm eligibility. Consequently, recruitment is a two-step process involving registration and randomisation.

Initial registration will involve all patients who have provided written informed consent. Patients will undergo investigations to confirm eligibility including a full blood count, biochemistry and renal function assessment, an electrocardiogram (ECG) and a pregnancy test (if applicable) to confirm that they satisfy the eligibility criteria specified in Table 1.

Once all other screening investigations are successfully completed and prior to meeting with the clinician and randomisation, two baseline QoL questionnaires (EORTC QLQ-C30[27] and EORTC QLQ-GINET21[28]) will be completed.

Patients identified as eligible, following the eligibility assessments, will be randomised. If more than 14 days have elapsed since the initial eligibility blood tests, these must be repeated prior to randomisation, to ensure that the patient remains eligible. Registration and randomisation will be

For peer review only - http://bmjopen.bmj.com/site/about/guidelines.xhtml

performed centrally using either the Leeds Clinical Trials Research Unit (CTRU) automated telephone or web-based system.

A minimisation program, which incorporates a random element, will be used for randomisation to ensure treatment groups are well balanced for the following characteristics:

- Hospital site
- Ki-67 marker (<55%, ≥55%)
- Eastern Co-operative Oncology Group (ECOG) performance status (0/1, 2),
- Presence of liver metastases (yes, no)
- Response to first-line platinum-based chemotherapy (resistant disease (progression ≤6 months from completion of platinum-based therapy), sensitive disease (progression >6 months from completion of platinum-based therapy), platinum intolerant).

Following randomisation, baseline assessments will be conducted. These will include; medical history, demographics, baseline symptoms, physical examination, vital signs, computed tomography (CT) scan (or magnetic resonance imaging (MRI) scan, if appropriate) of the thorax-abdomen-pelvis and staging within 28 days of starting trial treatment, one 10ml blood sample for local measurement of neuron-specific enolase and two 10ml blood samples for central translational research. Confirmation of availability of archival paraffin-embedded tissue for translational research will also be sought. An additional 10ml blood sample may be taken for mouse model development for consenting participants from The Christie NHS Foundation Trust.

Interventions

Nal-IRI (ONIVYDE®, Servier), folinic acid and 5-FU will be administered sequentially. The recommended dose and regimen of nal-IRI is 80 mg/m^2 body surface area (BSA) intravenously over 90 minutes (±10

For peer review only - http://bmjopen.bmj.com/site/about/guidelines.xhtml

minutes), followed by folinic acid as per local standard practice (recommended dose is 350 mg fixed dose), followed by 5-FU 2400 mg/m^2 BSA intravenously over 46 hours. Following cycle 1, subsequent doses will be administered every 14 days (+3 days/-1 day). Where it is not possible to administer nal-IRI due to toxicity, 5-FU/folinic acid can be administered as a monotherapy.

Docetaxel will be administered at a dose of 75 mg/m^2 BSA as an intravenous (IV) infusion over 60 minutes, or as per local standard practice. Following cycle 1, subsequent doses will be administered every 21 days (+3 days/-1 day).

Dosing may be postponed for up to 28 days from when it was due, to allow for (but not limited to) recovery from treatment-related toxicities, infection, or following patient request. In the event of a delay due to toxicity, a dose modification (see supplementary material) may be required at subsequent cycles following a dose delay. If a patient's dose is reduced due to toxicity, it will remain reduced for the duration of treatment. Patients who have already received two dose reductions and experience additional toxicities that would require further dose reduction should discontinue study medication. However, in the event that the participant is deriving clinical benefit and the treating clinician would prefer to continue treatment, an additional dose reduction may be permitted at the discretion of the Chief Investigator or delegate. If the toxicity recovery duration (to ≤Grade 2 CTCAE v5.0 or baseline) is more than 28 days, the participant should discontinue trial treatment. Participants who have prematurely discontinued treatment will continue to attend 8-weekly clinic visits for CT scans and have follow-up data collected, unless the participant withdraws consent for follow-up visits and further data collection.

All concurrent medical conditions and complications of the underlying malignancy will be treated at the discretion of the treating physician according to local standards of medical care. Participants can receive analgesics, antiemetics, antibiotics, antipyretics, and blood products as necessary. However, the use of warfarin-type anticoagulant therapies is not permitted.

For peer review only

Treatment cycle assessments

Participants on the nal-IRI/5-FU/folinic acid treatment arm will have 2-weekly treatment cycles. Participants on the docetaxel treatment arm will have 3-weekly treatment cycles.

Assessments carried out on the first day of each treatment cycle will include; laboratory assessments, clinical evaluation, vital signs, ECOG performance status, physical examination, details of concomitant medication and toxicity assessment (from cycle 2 onwards). Translational research blood samples and QoL questionnaires will be collected at 6-weekly intervals and at disease progression. A CT or MRI scan will be carried out 8-weekly (±7 days) from treatment start until disease progression or until 6 months after the last participant is randomised, whichever occurs first. Disease progression will be defined as the date of the CT or MRI scan that identifies disease progression. In the rare circumstances that disease progression is determined clinically and it is not appropriate to confirm it radiologically, the date of progression will be defined as the date of documented clinical disease progression.

Safety

Adverse events (AEs) and adverse reactions (ARs) will be collected on the first day of each treatment cycle from cycle 2 onwards. Serious adverse events (SAEs), serious adverse reactions (SARs) and suspected unexpected serious adverse reactions (SUSARs) will be collected from registration. All AEs, ARs and SAEs will be collected until 28 days after the last dose of trial treatment was administered; SARs and SUSARs will be collected until the end of the study.

Data collection

Data will be collected using paper case report forms (CRFs) and entered into a validated trial database by the CTRU, where data quality will be monitored. Automatic and manual validation of entered data will be conducted. Data items relating to the safety and rights of individual participants will be dealt

For peer review only - http://bmjopen.bmj.com/site/about/guidelines.xhtml

with as a priority. Data items required for the primary endpoint analysis will be manually checked at the CTRU. Missing data will be chased until it is either received or confirmed as not available at the trial analysis stage.

Statistical analysis

A full statistical analysis plan (SAP) will be written before any analysis is undertaken.

The primary analysis population will be defined as those who have received at least one dose of the protocol treatment. Individuals will be analysed according to the treatment that they received rather than that which they were randomised to receive. The QoL population is defined as any individual who returned at least one QoL questionnaire. Unless otherwise stated, the analysis will be conducted separately for each treatment group as per the primary analysis population.

All analyses will use a 5% significance level. The primary endpoint will be presented with a 1-sided CI, whilst secondary endpoints will be presented with 2-sided CIs. No formal interim analyses are scheduled to occur; hence, no statistical testing will take place until final analysis, which will occur once all randomised participants have reached the primary endpoint. Nevertheless, the Data Monitoring and Ethics Committee (DMEC) will receive full reports, at least annually and safety reports at least 6-monthly, to monitor participant safety and trial progress, and they may prematurely terminate the trial if necessary.

Primary endpoint analysis of the proportion of participants progression-free at 6 months post-randomisation will be calculated using exact methods. If the one-sided CI for either treatment from this analysis includes 15%, then that treatment will not be considered for a phase III trial.

Secondary endpoint analysis will include summary statistics and Kaplan-Meier survival curves for PFS and OS, summaries of the number and cause of deaths, and calculation of the ORR (defined as the

For peer review only - http://bmjopen.bmj.com/site/about/guidelines.xhtml

proportion of participants achieving at least a partial response (PR) within 6-months post randomisation).

Safety analyses will summarise AEs, ARs, SAEs, SARs, SUSARs, and pregnancies. Line listings of SAEs will be generated and will include details on expectedness, causality, relationship to the trial treatment and outcome.

Quality of life will be summarised using mean scores for each subscale and repeated measures models will be employed to investigate changes in health-related QoL over time for each treatment group, using the QoL population.

In the event that both treatment groups meet the specified threshold for the primary endpoint, and show a similar level of efficacy, toxicity and QoL data will inform which treatment to investigate in further research.

Summary statistics for the concentration of neuron-specific enolase at each time point will be estimated. The baseline concentration of neuron-specific enolase will be analysed to assess whether it is associated with response to treatment at 6 months post-randomisation, via an ordinal logistic regression model, adjusting for the stratification factors (excluding hospital site) and any appropriate interaction variables.

Exploratory analysis of the primary and selected secondary endpoints (PFS, OS and ORR) will be done using logistic or Cox regression, as appropriate. All models will be adjusted for the stratification factors (excluding hospital site). Subgroup analysis of the primary and selected secondary endpoints (as above) will include investigation of gender, age, Ki-67 value and morphology of NEC. All exploratory and subgroup analyses will be considered as hypothesis-generating rather than as confirmatory if significant differences are found. Further exploratory and subgroup analyses beyond that described may be undertaken.

For peer review only

Trial monitoring

A Trial Monitoring Plan will be developed by the Trial Management Group (TMG) and agreed by the Trial Steering Committee (TSC), based on the trial risk assessment. The TMG, comprising the Chief Investigator, CTRU team, other key trial staff, a nursing representative and a patient and public involvement (PPI) representative will be assigned responsibility for the clinical setup, on-going management, promotion of the trial and the interpretation and publishing of the results. The TSC and DMEC will provide independent oversight of the study and will be responsible for monitoring the study conduct. The TSC, comprising a statistician, an oncologist and a PPI representative will provide overall supervision of the trial. The DMEC, comprised of two gastroenterologists, an oncologist (all with experience in the treatment of patients with NENs) and a statistician, will review the safety and ethics of the study alongside the trial progress, as overseen by the TSC. The DMEC will review confidential safety reports at least 6-monthly and the DMEC and TSC will meet separately, at least annually, to discuss trial progress.

Patient and public involvement

Patient and public involvement representatives are involved in the design and overall direction of the trial through their roles in the TMG and the TSC. As part of the TMG, the PPI representative has been involved in protocol development and the preparation of the patient information and informed consent trial documentation. As part of the TSC, the PPI representative will provide advice regarding trial design and conduct, and will be involved in monitoring trial progress and patient safety.

ETHICS AND DISSEMINATION

The NET-02 trial opened to recruitment on 16th November 2018. At the time of submission, 12 centres out of 16 are open to recruitment, and 17 participants have been randomised into the trial. The trial

For peer review only - http://bmjopen.bmj.com/site/about/guidelines.xhtml

is currently adhering to version 3.0 of the protocol (approved 20th September 2018), with all sites opening to this version of the protocol. The trial is sponsored by The Christie NHS Foundation Trust, coordinated by Leeds CTRU and funded by Servier (unrestricted grant). The trial is registered on the International Standard Randomised Controlled Trial registry (ISRCTN: 10996604), European Clinical Trials Database (EudraCT: 2017-002453-11) and ClinicalTrials.gov: NCT03837977.

This study has ethical approval from the Greater Manchester Central Research Ethics Committee (Reference No. 18/NW/0031) and clinical trial authorisation from the Medicine and Healthcare Products Regulatory Agency. The trial will be conducted in accordance with Good Clinical Practice. Trial results will be published in peer-reviewed journals and will be reported in line with the Consolidated Standards of Reporting Trials (CONSORT) guidelines.[31] Authorship will be decided according to the International Committee of Medical Journal Editors (ICMJE) criteria for authorship.[32] All publications will be reviewed by the Sponsor and funder prior to publication. To maintain the scientific integrity of the trial, data will not be released prior to the first publication of the analysis of the primary endpoint, for either trial publication or oral presentation purposes, without the permission of the Sponsor and TSC. Research results will also be uploaded to the EU Clinical Trials Register.

All information collected during the course of the trial will be kept strictly confidential. Information will be held securely at the CTRU. The CTRU will comply with all aspects of the General Data Protection Regulation (GDPR) 2018.[33] The trial staff at the participating sites will be responsible for ensuring that any data or documentation sent to the CTRU is appropriately anonymised. At the end of the trial, data will be securely archived in line with the Sponsor's procedures for a minimum of 15 years. Data held by the CTRU will be archived in the Sponsor archive facility, and site data and documents will be archived at the sites. Following authorisation from the Sponsor, arrangements for confidential destruction will then be made.

For peer review only - http://bmjopen.bmj.com/site/about/guidelines.xhtml

AUTHOR CONTRIBUTIONS:

Conception and design of the NET-02 trial: JS, DAC, HH, JWV and MMN. Development of the protocol and patient information sheet: JS, DAC, HH, DS, OF, TM, JWV and MMN. Writing of manuscript: ZC, JS, DAC, and MMN. Review of manuscript: EB, JW, NR, OF, JC, RS, IC, LW, AL, RAH, MW, DS, TM and HH.

All authors have read and approved the final manuscript.

ACKNOWLEDGEMENTS:

Thanks to Dr Alison Backen (Project Manager, The Christie NHS Foundation Trust) for her contribution to initial protocol development.

FUNDING:

This research is investigator-initiated and funded by an unrestricted educational grant from Servier (grant reference number 016-34263). This work was also supported by Core Clinical Trials Unit Infrastructure from Cancer Research UK (C7852/A25447).

SPONSOR: The Christie NHS Foundation Trust, Wilmslow Road, Manchester, M20 4BX, United Kingdom. Sponsor reference: CFTSp116

COMPETING INTERESTS:

JW reports grants and personal fees from AstraZeneca, grants and personal fees from Sanofi-Genzyme, personal fees and non-financial support from Celgene, personal fees from Eisai, personal

For peer review only

fees and non-financial support from Ipsen, personal fees and non-financial support from Novartis, non-financial support from Imaging Equipment Ltd, outside the submitted work.

IC reports advisory role for Eli-Lilly, Bristol Meyers Squibb, MSD, Bayer, Roche, Merck-Serono, Five Prime Therapeutics, AstraZeneca, Oncologie International, Pierre Fabre; research funding from Eli-Lilly, Janssen-Cilag, Sanofi Oncology, Merck-Serono; honorarium from Eli-Lilly.

AL received travel and educational support from Ipsen, Pfizer, Bayer, AAA, Sirtex Medical, Novartis, Mylan and Delcath Systems; speaker honoraria from Merck, Pfizer, Ipsen and Incyte; advisory honoraria from EISAI and Nutricia; she is a member of the Knowledge Network and NETConnect Initiatives funded by Ipsen.

DS reports personal fees from MSD, personal fees and non-financial support from EISAI, personal fees and non-financial support from Ipsen, personal fees from Bayer, non-financial support from Mina Therapeutics, personal fees from Pfizer, personal fees from Novartis, outside the submitted work.

TM reports grants from Bayer, grants from BTG, personal fees from BMS, personal fees from EISAI, personal fees from AstraZeneca, personal fees from Tarveda , personal fees from Ipsen, personal fees from MSD, outside the submitted work.

DAC reports grants and non-financial support from Servier, during the conduct of the study.

HH reports grants and non-financial support from Servier, during the conduct of the study.

JWV reports Consulting or Advisory role for Ipsen, Novartis, AstraZeneca, Merck, Delcath Systems, Agios, Pfizer, PCI Biotech, Incyte, Keocyt, QED, Pieris Pharmaceuticals, Genoscience Pharma, Mundipharma EDO; Honoraria from Ipsen; and Speakers' Bureau for Novartis, Ipsen, NuCana and Imaging Equipment Limited.

MMN has received research grant support from Servier, Ipsen and NuCana. She has received travel and accommodation support from Bayer and Ipsen and speaker honoraria from Pfizer, Ipsen and NuCana. She has served on advisory boards for Celgene, Ipsen, Sirtex and Baxalta.

For peer review only - http://bmjopen.bmj.com/site/about/guidelines.xhtml

For peer review only

EXCLUSIVE LICENCE:

I, the Submitting Author has the right to grant and does grant on behalf of all authors of the Work (as defined in the below author licence), an exclusive licence and/or a non-exclusive licence for contributions from authors who are: i) UK Crown employees; ii) where BMJ has agreed a CC-BY licence shall apply, and/or iii) in accordance with the terms applicable for US Federal Government officers or employees acting as part of their official duties; on a worldwide, perpetual, irrevocable, royalty-free basis to BMJ Publishing Group Ltd ("BMJ") its licensees and where the relevant Journal is co-owned by BMJ to the co-owners of the Journal, to publish the Work in BMJ Open and any other BMJ products and to exploit all rights, as set out in our licence.

The Submitting Author accepts and understands that any supply made under these terms is made by BMJ to the Submitting Author unless you are acting as an employee on behalf of your employer or a postgraduate student of an affiliated institution which is paying any applicable article publishing charge ("APC") for Open Access articles. Where the Submitting Author wishes to make the Work available on an Open Access basis (and intends to pay the relevant APC), the terms of reuse of such Open Access shall be governed by a Creative Commons licence – details of these licences and which Creative Commons licence will apply to this Work are set out in our licence referred to above.

For peer review only

REFERENCES

1. Leoncini E, Boffetta P, Shafir M, et al. Increased incidence trend of low-grade and high-grade neuroendocrine neoplasms. Endocrine 2017;**58**(2):368-79.
2. Dasari A, Mehta K, Byers LA, et al. Comparative study of lung and extrapulmonary poorly differentiated neuroendocrine carcinomas: A SEER database analysis of 162,983 cases. Cancer 2018;**124**(4):807-15.
3. Korse CM, Taal BG, van Velthuysen MLF, et al. Incidence and survival of neuroendocrine tumours in the Netherlands according to histological grade: Experience of two decades of cancer registry. European Journal of Cancer 2013;**49**(8):1975-83.
4. Brenner B, Tang LH, Shia J, et al. Small Cell Carcinomas of the Gastrointestinal Tract: Clinicopathological Features and Treatment Approach. Seminars in Oncology 2007;**34**(1):43-50.
5. Bosman FT, Carneiro F, Hruban RH, et al. WHO classification of tumours of the digestive system, 4th edition. WHO Press 2010.
6. Moertel CG, Kvols LK, O'Connell MJ, et al. Treatment of neuroendocrine carcinomas with combined etoposide and cisplatin. Evidence of major therapeutic activity in the anaplastic variants of these neoplasms. Cancer 1991;**68**(2):227-32.
7. Hentic O, Hammel P, Couvelard A, et al. FOLFIRI regimen: an effective second-line chemotherapy after failure of etoposide-platinum combination in patients with neuroendocrine carcinomas grade 3. Endocrine Related Cancer 2012;**19**(6):751-57.
8. Welin S, Sorbye H, Sebjornsen S, et al. Clinical effect of temozolomide-based chemotherapy in poorly differentiated endocrine carcinoma after progression on first-line chemotherapy. Cancer 2011;**117**(20):4617-22.
9. Sorbye H, Welin S, Langer SW, et al. Predictive and prognostic factors for treatment and survival in 305 patients with advanced gastrointestinal neuroendocrine carcinoma (WHO G3): The NORDIC NEC study. Annals of Oncology 2013;**24**(1):152-60.
10. Hadoux J, Guigay J, Malka D, et al. Oxaliplatin-based chemotherapy for grade 3 neuroendocrine carcinoma after failure of platinum-based chemotherapy. European Neuro Endocrine Tumour Society 2013;**Abstract J2**.
11. Olsen IH, Knigge U, Federspiel B, et al. Topotecan Monotherapy in Heavily Pretreated Patients with Progressive Advanced Stage Neuroendocrine Carcinomas. Journal of Cancer 2014;**5**(8):628-32.
12. Heetfeld M, Chougnet CN, Olsen IH, et al. Characteristics and treatment of patients with G3 gastroenteropancreatic neuroendocrine neoplasms. Endocrine-Related Cancer 2015;**22**(4):657-64.
13. Olsen IH, Sørensen JB, Federspiel B, et al. Temozolomide as Second or Third Line Treatment of Patients with Neuroendocrine Carcinomas. The Scientific World Journal 2012;**2012**:1-4.
14. Yamaguchi T, Machida N, Morizane C, et al. Multicenter retrospective analysis of systemic chemotherapy for advanced neuroendocrine carcinoma of the digestive system. Cancer Sci 2014;**105**(9):1176-81.
15. McNamara MG, Frizziero M, Jacobs T, et al. Second-line treatment in patients (pts) with advanced extra-pulmonary poorly differentiated neuroendocrine carcinoma (EP-PD-NEC): A systematic review and meta-analysis. Neuroendocrinology 2019;**108**(Suppl 1):1-273.
16. Irinotecan hydrochloride Summary of Product Characteristics. https://www.medicines.org.uk/emc/product/5794/smpc#PHARMACOLOGICAL_PROPS (accessed 23 March 2016).
17. ONIVYDE 5 mg/ml concentrate for solution for infusion Summary of Product Characteristics. https://www.medicines.org.uk/emc/product/9200#PHARMACOLOGICAL_PROPS (accessed 01 July 2019).

For peer review only - http://bmjopen.bmj.com/site/about/guidelines.xhtml

18. Camptosar (irinotecan) U.S. Package Insert. http://www.accessdata.fda.gov/drugsatfda_docs/label/2014/020571s048lbl.pdf (accessed 23 March 2016).
19. Baker JH, Lam J, Kyle AH, et al. Irinophore C, a novel nanoformulation of irinotecan, alters tumor vascular function and enhances the distribution of 5-fluorouracil and doxorubicin. Clin Cancer Res 2008;**14**(22):7260-71.
20. Verreault M, Strutt D, Masin D, et al. Vascular normalization in orthotopic glioblastoma following intravenous treatment with lipid-based nanoparticulate formulations of irinotecan (Irinophore C), doxorubicin (Caelyx(R)) or vincristine. BMC Cancer 2011;**11**:124.
21. Chiang NJ, Chao TY, Hsieh RK, et al. A phase I dose-escalation study of PEP02 (irinotecan liposome injection) in combination with 5-fluorouracil and leucovorin in advanced solid tumors. BMC Cancer 2016;**16**(1):907.
22. Chen LT, Von Hoff DD, Li CP, et al. Expanded analyses of napoli-1: Phase 3 study of MM-398 (nal-IRI), with or without 5-fluorouracil and leucovorin, versus 5-fluorouracil and leucovorin, in metastatic pancreatic cancer (mPAC) previously treated with gemcitabine-based therapy. Journal of Clinical Oncology 2015;**33**(3_suppl):234-34.
23. NCCN guidelines: Small Cell Lung Cancer. https://www.nccn.org/professionals/physician_gls/pdf/sclc.pdf (accessed 05 Aug 2019).
24. NCCN guidelines: Non-Small Cell Lung Cancer. https://www.nccn.org/professionals/physician_gls/pdf/nscl.pdf (accessed 09 Sep 2019).
25. Fossella FV. Docetaxel for previously treated non-small-cell lung cancer. Oncology (Williston Park) 2002;**16**(6 Suppl 6):45-51.
26. Eisenhauer EA, Therasse P, Bogaerts J, et al. New response evaluation criteria in solid tumours: Revised RECIST guideline (version 1.1). European Journal of Cancer 2009;**45**(2):228-47.
27. Groenvold M, Klee MC, Sprangers MA, et al. Validation of the EORTC QLQ-C30 quality of life questionnaire through combined qualitative and quantitative assessment of patient-observer agreement. Journal of clinical epidemiology 1997;**50**(4):441-50.
28. Yadegarfar G, Friend L, Jones L, et al. Validation of the EORTC QLQ-GINET21 questionnaire for assessing quality of life of patients with gastrointestinal neuroendocrine tumours. British journal of cancer 2013;**108**(2):301-10.
29. Simon R, Wittes RE, Ellenberg SS. Randomized phase II clinical trials. Cancer Treat Rep 1985;**69**(12):1375-81.
30. A'Hern RP. Sample size tables for exact single-stage phase II designs. Stat Med 2001;**20**(6):859-66.
31. Consolidated Standards of Reporting Trials Guidelines. http://www.consort-statement.org/ (accessed 07 Dec 2018).
32. International Committee of Medical Journal Editors: Defining the Role of Authors and Contributors. http://www.icmje.org/recommendations/browse/roles-and-responsibilities/defining-the-role-of-authors-and-contributors.html (accessed 02 Jul 2019).
33. Information Commissioner's Office: Guide to the General Data Protection Regulation (GDPR). https://ico.org.uk/for-organisations/guide-to-data-protection/guide-to-the-general-data-protection-regulation-gdpr/ (accessed 02 Jul 2019).

For peer review only - http://bmjopen.bmj.com/site/about/guidelines.xhtml

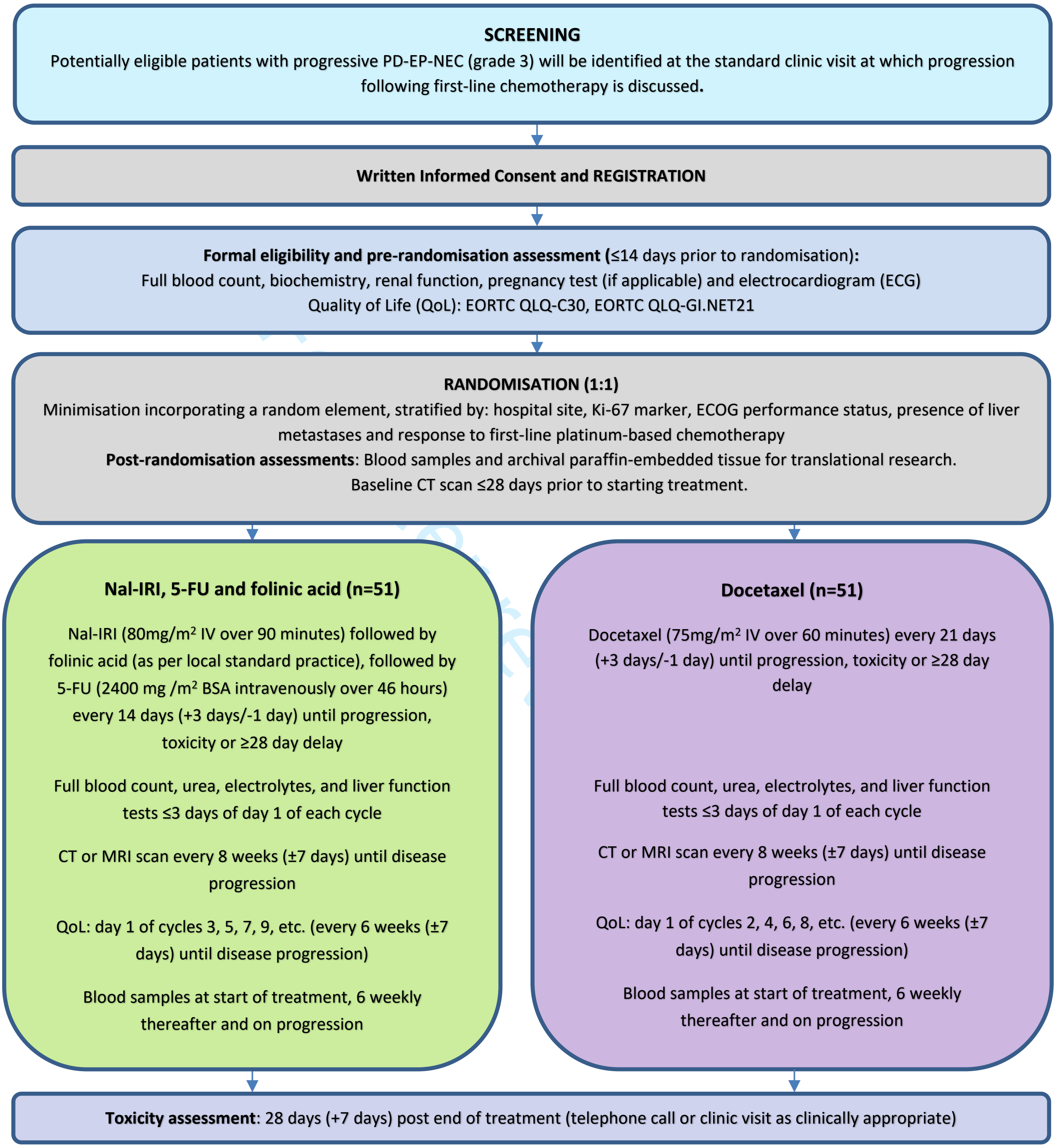
SCREENING
Potentially eligible patients with progressive PD-EP-NEC (grade 3) will be identified at the standard clinic visit at which progression following first-line chemotherapy is discussed.
Written Informed Consent and REGISTRATION
Formal eligibility and pre-randomisation assessment (≤14 days prior to randomisation):
Full blood count, biochemistry, renal function, pregnancy test (if applicable) and electrocardiogram (ECG)
Quality of Life (QoL): EORTC QLQ-C30, EORTC QLQ-GI.NET21
RANDOMISATION (1:1)
Minimisation incorporating a random element, stratified by: hospital site, Ki-67 marker, ECOG performance status, presence of liver metastases and response to first-line platinum-based chemotherapy
Post-randomisation assessments: Blood samples and archival paraffin-embedded tissue for translational research.
Baseline CT scan ≤28 days prior to starting treatment.
Nal-IRI, 5-FU and folinic acid (n=51)
Nal-IRI (80mg/m² IV over 90 minutes) followed by folinic acid (as per local standard practice), followed by 5-FU (2400 mg /m² BSA intravenously over 46 hours) every 14 days (+3 days/-1 day) until progression, toxicity or ≥28 day delay
Full blood count, urea, electrolytes, and liver function tests ≤3 days of day 1 of each cycle
CT or MRI scan every 8 weeks (±7 days) until disease progression
QoL: day 1 of cycles 3, 5, 7, 9, etc. (every 6 weeks (±7 days) until disease progression)
Blood samples at start of treatment, 6 weekly thereafter and on progression
Docetaxel (n=51)
Docetaxel (75mg/m² IV over 60 minutes) every 21 days (+3 days/-1 day) until progression, toxicity or ≥28 day delay
Full blood count, urea, electrolytes, and liver function tests ≤3 days of day 1 of each cycle
CT or MRI scan every 8 weeks (±7 days) until disease progression
QoL: day 1 of cycles 2, 4, 6, 8, etc. (every 6 weeks (±7 days) until disease progression)
Blood samples at start of treatment, 6 weekly thereafter and on progression
Toxicity assessment: 28 days (+7 days) post end of treatment (telephone call or clinic visit as clinically appropriate)

Supplementary material

Contents

Supplementary material 1
1. TRIAL SITES 2
2. DOSE MODIFICATIONS 3
2.1 Liposomal irinotecan (nal-IRI) dose modifications........ 3
2.2 5-Fluorouracil (5-FU) and folinic acid dose modifications 5
2.2.1 Haematological toxicities: 5-FU dose modifications........ 5
2.2.2 Non-haematological toxicities: 5-FU dose modifications 5
2.2.3 Other toxicity requiring special attention........ 6
2.3 Docetaxel dose modifications 6
2.4 Management of infusion reactions 8

For peer review only

1. TRIAL SITES

Table 1 list the trial sites, which are taking part in the NET-02 trial, indicating if they are open to recruitment or are in set-up. The principal investigator (PI) of each site is listed and for sites that are open to recruitment, the number or patients randomised up to the point of submission are provided.

Table 1 | Trial sites

Site status	Site Name	PI Name	Number of patients randomised
Open	Christie Hospital	Dr Mairéad McNamara	8
	Weston Park Hospital	Dr Jon Wadsley	2
	Beatson	Professor Nick Reed	2
	Hammersmith Hospital	Dr Rohini Sharma	1
	Royal Free Hospital	Dr Daniel Krell	0
	Royal Marsden Hospital	Dr Ian Chau	1
	Western General Hospital	Dr Lucy Wall	1
	The Clatterbridge Cancer Centre	Dr Olusola Faluyi	1
	University Hospital Southampton	Dr Judith Cave	1
	Velindre Cancer Centre	Dr Carys Morgan	0
	Guy's Hospital	Dr Debashis Sarker	0
	Newcastle	Dr Jane Margetts	0
In set-up	University Hospital Coventry	Dr Sharmila Sothi	n/a
	Belfast	Dr Martin Eatock	n/a
	St James University Hospital	Dr Alan Anthoney	n/a
	Cheltenham General Hospital	Dr David Farrugia	n/a

2. DOSE MODIFICATIONS

2.1 Liposomal irinotecan (nal-IRI) dose modifications

For Grade 1 and 2 toxicities, no dose modifications are required. In the event of Grade 3 or 4 toxicity, the doses of liposomal irinotecan (nal-IRI) and 5-fluorouracil (5-FU) must be reduced and subsequent doses of nal-IRI and 5-FU must continue to be adjusted as indicated in Table 2. All dose modifications must be based on the worst preceding toxicity.

Table 2 | Dose modifications for nal-IRI and 5-FU and folinic acid, for Grade 3-4 toxicities.

	Toxicity<br>CTCAE Grade (value)	Dose Adjustment
**Haematological toxicities**	**Neutropenia**<br>Grade 3 or Grade 4<br>(<1000/mm^3: <1x10^9/L)<br>Or neutropenic fever	**A new cycle of therapy should not begin until the absolute neutrophil count is ≥1.5x10^9/L (dose modifications below are for subsequent treatments, if grade 3 or 4 neutropenia is recorded on day 1 of a cycle or neutropenic fever is experienced during a cycle)***<br>**First occurrence** Reduce nal-IRI dose to 60 mg/m^2<br>Reduce 5-FU dose by 25% (1800 mg/m^2)<br>**Second occurrence** Reduce nal-IRI dose to 50 mg/m^2<br>Reduce 5-FU dose by 25% (1350 mg/m^2)<br>**Third occurrence** Discontinue treatment
	**Thrombocytopenia**<br>**Leukopenia**<br>Grade 3 or 4	**A new cycle of therapy should not begin until the platelet count is ≥100x10^9/L**<br>Dose modifications for grade 3 or 4 thrombocytopenia are the same as recommended for neutropenia above for first, second and third recurrence.
**Non-haematological toxicities**	**Diarrhoea**<br>Grade 3 or 4<br>(≥7 stools per day pre-treatment)	**A new cycle of therapy should not begin until diarrhoea resolves to ≤Grade 1** (2-3 stools/day more than pre-treatment frequency) **(Dose modifications below are for subsequent treatments)**<br>**First occurrence** Reduce nal-IRI dose to 60 mg/m^2<br>Reduce 5-FU dose by 25% (1800 mg/m^2)<br>**Second occurrence** Reduce nal-IRI dose to 50 mg/m^2<br>Reduce 5-FU dose by 25% (1350 mg/m^2)

For peer review only

	Toxicity<br>CTCAE Grade (value)	Dose Adjustment
		**Third occurrence** Discontinue treatment
	**Nausea/vomiting**<br>Grade 3 or 4 despite optimal antiemetic therapy	**A new cycle of therapy should not begin until nausea/vomiting resolves to ≤Grade 1 or baseline (Dose modifications below are for subsequent treatments)**<br>**First occurrence** Optimise antiemetic therapy<br>Reduce nal-IRI dose to 60 mg/m^2<br>**Second occurrence** Optimise antiemetic therapy<br>Reduce nal-IRI dose to 50 mg/m^2<br>**Third occurrence** Discontinue treatment
	**Hepatic, renal, respiratory or other toxicities**<br>Grade 3 or 4<br>(asthenia and grade 3 anorexia do not require dose adjustment and also excluding grade ≥3 ALT/AST which resolve to baseline within 7 days and grade ≥3 toxicities which following case causality assessment are not in the category of 'Certain', 'Probable' or 'Possible' and as such are not related to study treatment, or which are not considered a clinically-significant toxicity)	**A new cycle of therapy should not begin until the adverse reaction resolves to ≤Grade 1 (Dose modifications below are for subsequent treatments)**<br>**First occurrence** Reduce nal-IRI dose to 60 mg/m^2<br>Reduce 5-FU dose by 25% (1800 mg/m^2)<br>**Second occurrence** Reduce nal-IRI dose to 50 mg/m^2<br>Reduce 5-FU dose by 25% (1350 mg/m^2)<br>**Third occurrence** Discontinue treatment

* Prophylactic use of granulocyte colony-stimulating factor (G-CSF) can be considered prior to dose modification in those patients who have had at least one episode of grade 3 or 4 neutropenia or neutropenic fever while receiving therapy or have had documented grade 3 or 4 neutropenia or neutropenic fever while receiving prior anti-neoplastic therapy.
CTCAE: Common Terminology Criteria for Adverse Events, ALT: alanine aminotransferase, AST: aspartate aminotransferase.

For peer review only

2.2 5-Fluorouracil (5-FU) and folinic acid dose modifications

Dose modifications for 5-FU are provided below. No dose adjustments for toxicity are required for folinic acid. Folinic acid must be given immediately prior to each 5-FU dose; hence, if the 5-FU dose is held, folinic acid dose should be held as well. If the dosing of nal-IRI needs to be withheld, then the 5-FU/folinic acid in the combination can be administered as monotherapy. In case a patient experiences an infusion reaction, either institutional guidelines or the guidelines provided for nal-IRI infusion reaction management (section 2.4) should be used.

2.2.1 Haematological toxicities: 5-FU dose modifications

Absolute neutrophil count (ANC) and platelet count should be measured locally no more than 3 days prior to day 1 of each treatment cycle. Treatment should only proceed if;

- ANC $\geq 1.5 \times 10^9$/L
- Platelet count $\geq 100 \times 10^9$/L

In the event of haematological toxicity, treatment should be delayed (up to 28 days) to allow sufficient time for recovery. On recovery, treatment should be administered according to the guidelines provided in Table 2.

2.2.2 Non-haematological toxicities: 5-FU dose modifications

Treatment should be delayed until all clinically significant Grade 3 or 4 non-haematological toxicities resolve to Grade 1 or baseline. If delays are greater than 28 days for toxicity, the participant should be withdrawn from trial treatment. Dose adjustments of other 5-FU-related toxicities are provided in Table 3. Asthenia, grade 3 anorexia and grade ≥3 toxicities which following case causality assessment are not in the category of 'Certain', 'Probable' or 'Possible' and as such are not related to study treatment, or which are not considered a clinically-significant toxicity, do not require dose modifications.

For peer review only

Table 3 | 5-FU dose modifications for other non-haematological toxicities

Worst toxicity CTCAE grade	5-FU dose for next cycle[a]
Grade 1 or 2	100% of previous dose, except for Grade 2 hand-foot syndrome, Grade 2 cardiac toxicity, or any grade neurocerebellar toxicity
Grade 2 hand-foot syndrome	Reduce dose by 25%[b]
Any grade neurocerebellar or ≥ Grade 2 cardiac toxicity	Discontinue therapy
Grade 3 or 4	Reduce dose by 25%[b], except for Grade 3 or 4 hand-foot syndrome
Grade 4 or 4 hand-foot syndrome	Discontinue therapy

[a] All dose modifications must be based on the worst preceding toxicity.

[b] Participants who require more than 2 dose reductions should be withdrawn from trial treatment unless agreed with the Chief Investigator or delegate.

CTCAE: Common Terminology Criteria for Adverse Events, 5-FU; 5-Fluorouracil.

For peer review only

2.2.3 Other toxicity requiring special attention

Corrected QT interval (QTc) prolongation that occurs in the setting of diarrhoea-induced electrolyte imbalance should be treated with appropriate electrolyte repletion. Once the underlying abnormality is corrected and the electrocardiogram (ECG) abnormalities have reversed, treatment may continue under careful monitoring, and with appropriate dose modification for diarrhoea as per local standard of care practice.

2.3 Docetaxel dose modifications

Neutrophil and platelet count should be measured locally no more than 3 days prior to day 1 of each treatment cycle. Treatment should only proceed if:

- ANC ≥ 1.5×10^9/L
- Platelet count ≥100×10^9/L
- Bilirubin ≤1.5 x upper limit of normal (ULN)
- Alanine aminotransferase (ALT) and/or Aspartate aminotransferase (AST) ≤2.5 x ULN (in absence of liver metastasis) or ≤5 x ULN (in presence of liver metastasis)

In the event of haematological toxicity, treatment should be delayed (up to 28 days) to allow sufficient time for recovery. Guidelines for docetaxel dose modifications are provided in Table 4.

Table 4 | Dose reductions for docetaxel toxicities

Toxicity	Severity	Management
**Hypersensitivity**	Grade 3/Grade 4	Administration of appropriate medication (see below).
**Neutropenia**	Day 1 neutrophil count<br><1500/mm^3: <1.5x10^9/L	Stop treatment until neutrophils recovers to at least 1.5x10^9/L. If neutrophils <1.5x10^9/L for ≤7 days, restart docetaxel at full dose (75mg/m^2). If neutrophils <1.5x10^9/L for >7 days, restart docetaxel at 55mg/m^2 or next lowest dose level (40mg/m^2) if already reduced.*
	Febrile neutropenia OR prolonged Grade 4 neutropenia (Neutrophil count <500/mm^3: <0.5x10^9/L for 7 days or more)	Stop treatment until neutrophils ≥ 1.5x10^9/L. Restart drug at 55mg/m^2 or next lowest dose level (40mg/m^2) if already reduced.*
**Neuropathy**	Grade 3/Grade 4	Stop docetaxel treatment.
**Thrombocytopenia**	Platelet count <100 x 10^9/L	Stop treatment until platelets ≥100x^9/L. Restart drug at full dose (75mg/m^2).
	Platelet count <50 x 10^9/L (Grade 3/Grade 4)	Stop treatment until platelets ≥100x^9/L. Restart drug at 55mg/m^2 or next lowest dose level (40mg/m^2) if already reduced.
**Hepatic Dysfunction**	Bilirubin >1.5 ULN<br>ALT/AST>2.5 x ULN (in absence of liver metastasis), >5 x ULN (in presence of liver metastasis)	Stop docetaxel until parameters recover to baseline levels. Restart drug at 55mg/m^2 or next lowest dose level (40mg/m^2) if already reduced.
**Cutaneous reaction**	Grade 2	Stop treatment until recovery to Grade 1 or better. Restart drug at full dose (75mg/m^2).
	Severe or cumulative<br>(Grade 3/Grade 4)	Stop treatment until recovery (Grade 1 or better). Restart drug at 55 mg/m^2 or next

For peer review only

Toxicity	Severity	Management
		lowest dose level (40mg/m2) if already reduced.
**Other non-haematological toxicity (excluding grade ≥3 toxicities which following case causality assessment are not in the category of 'Certain', 'Probable' or 'Possible' and as such is not related to study treatment, or which are not considered a clinically-significant toxicity)**	Grade 3/Grade 4	Stop docetaxel until parameters recover to baseline levels. Restart drug at 55mg/m^2 or next lowest dose level (40mg/m^2) if already reduced.

* Prophylactic use of granulocyte colony-stimulating factor (G-CSF) can be considered prior to dose modification in those patients who have had at least one episode of grade 3 or 4 neutropenia or neutropenic fever while receiving therapy or have had documented grade 3 or 4 neutropenia or neutropenic fever while receiving prior anti-neoplastic therapy.
ULN: upper limit of normal, ALT: alanine aminotransferase, AST: aspartate aminotransferase.

2.4 Management of infusion reactions

The guidelines described in this section can be followed in case of infusion reactions. Infusion reactions will be defined according to the National Cancer Institute Common Terminology Criteria for Adverse Events (CTCAE) (Version 5.0) definitions of an allergic reaction or anaphylaxis. Institutional policies or the following treatment guidelines shall be used for the management of infusion reactions.

Grade 1

- Slow infusion rate by 50%.
- Monitor patient every 15 minutes for worsening of condition.
- Future infusions may be administered at a reduced rate (e.g. over 120 minutes for nal-IRI), at the discretion of the Investigator.

Grade 2

- Stop infusion.

For peer review only

- Administer diphenhydramine hydrochloride 50 mg intravenously (IV) (or similar), acetaminophen 650 mg (or similar) orally, and oxygen.
- Resume infusion at 50% of the prior rate once infusion reaction has resolved.
- Monitor patient every 15 minutes for worsening of condition.
- For all subsequent infusions, pre-medicate with diphenhydramine hydrochloride 50 mg IV (or similar), dexamethasone 10 mg IV, and acetaminophen 650 mg (or similar) orally.
- Future infusions may be administered at a reduced rate (e.g. over 120 minutes for nal-IRI), at the discretion of the Investigator.

Grade 3

- Stop infusion and disconnect infusion tubing from patient.
- Administer diphenhydramine hydrochloride 50 mg IV (or similar), dexamethasone 10 mg IV, bronchodilators for bronchospasm, and other medications or oxygen as medically necessary.
- No further treatment will be permitted.

Grade 4

- Stop the infusion and disconnect infusion tubing from patient.
- Administer epinephrine (adrenaline), bronchodilators or oxygen as indicated for bronchospasm.
- Administer diphenhydramine hydrochloride 50 mg IV (or similar), dexamethasone 10 mg IV and other medications as medically necessary.
- Consider hospital admission for observation.
- No further treatment will be permitted.

For peer review only

For patients who experience a Grade 1 or Grade 2 infusion reaction, future infusions may be administered at a reduced rate (over 120 minutes), at the discretion of the treating physician. For patients who experience a second grade 1 or 2 infusion reaction, administer dexamethasone 10 mg IV. All subsequent infusions should be pre-medicated with diphenhydramine hydrochloride 50 mg IV (or similar), dexamethasone 10 mg IV, and acetaminophen 650 mg orally (or similar).

For peer review only

Reporting checklist for protocol of a clinical trial.

Based on the SPIRIT guidelines.

Instructions to authors

Complete this checklist by entering the page numbers from your manuscript where readers will find each of the items listed below.

Your article may not currently address all the items on the checklist. Please modify your text to include the missing information. If you are certain that an item does not apply, please write "n/a" and provide a short explanation.

Upload your completed checklist as an extra file when you submit to a journal.

In your methods section, say that you used the SPIRITreporting guidelines, and cite them as:

Chan A-W, Tetzlaff JM, Altman DG, Laupacis A, Gøtzsche PC, Krleža-Jerić K, Hróbjartsson A, Mann H, Dickersin K, Berlin J, Doré C, Parulekar W, Summerskill W, Groves T, Schulz K, Sox H, Rockhold FW, Rennie D, Moher D. SPIRIT 2013 Statement: Defining standard protocol items for clinical trials. Ann Intern Med. 2013;158(3):200-207

		Reporting Item	Page Number
**Administrative information**			
Title	#1	Descriptive title identifying the study design, population, interventions, and, if applicable, trial acronym	1

For peer review only

Trial registration	#2a	Trial identifier and registry name. If not yet registered, name of intended registry	2, 19
Trial registration: data set	#2b	All items from the World Health Organization Trial Registration Data Set	Can be accessed via ISRCTN website using the ISRCTN on page 2
Protocol version	#3	Date and version identifier	19
Funding	#4	Sources and types of financial, material, and other support	19, 20
Roles and responsibilities: contributorship	#5a	Names, affiliations, and roles of protocol contributors	1, 20
Roles and responsibilities: sponsor contact information	#5b	Name and contact information for the trial sponsor	20
Roles and responsibilities: sponsor and funder	#5c	Role of study sponsor and funders, if any, in study design; collection, management, analysis, and interpretation of data; writing of the report; and the decision to submit the report for publication, including whether they will have ultimate authority over any of these activities	19

For peer review only

Roles and responsibilities: committees	#5d	Composition, roles, and responsibilities of the coordinating centre, steering committee, endpoint adjudication committee, data management team, and other individuals or groups overseeing the trial, if applicable (see Item 21a for data monitoring committee)	18
**Introduction**			4-7
Background and rationale	#6a	Description of research question and justification for undertaking the trial, including summary of relevant studies (published and unpublished) examining benefits and harms for each intervention	
Background and rationale: choice of comparators	#6b	Explanation for choice of comparators	5-6
Objectives	#7	Specific objectives or hypotheses	7-8
Trial design	#8	Description of trial design including type of trial (eg, parallel group, crossover, factorial, single group), allocation ratio, and framework (eg, superiority, equivalence, non-inferiority, exploratory)	8-9
**Methods: Participants,**			

For peer review only

**interventions, and outcomes**			
Study setting	#9	Description of study settings (eg, community clinic, academic hospital) and list of countries where data will be collected. Reference to where list of study sites can be obtained	12
Eligibility criteria	#10	Inclusion and exclusion criteria for participants. If applicable, eligibility criteria for study centres and individuals who will perform the interventions (eg, surgeons, psychotherapists)	9-11
Interventions: description	#11a	Interventions for each group with sufficient detail to allow replication, including how and when they will be administered	13-14
Interventions: modifications	#11b	Criteria for discontinuing or modifying allocated interventions for a given trial participant (eg, drug dose change in response to harms, participant request, or improving / worsening disease)	14
Interventions: adherance	#11c	Strategies to improve adherence to intervention protocols, and any procedures for monitoring adherence (eg, drug tablet return; laboratory tests)	N/A – treatment administered in hospital
Interventions: concomitant care	#11d	Relevant concomitant care and interventions that are permitted or prohibited during the trial	14

For peer review only

Outcomes	#12	Primary, secondary, and other outcomes, including the specific measurement variable (eg, systolic blood pressure), analysis metric (eg, change from baseline, final value, time to event), method of aggregation (eg, median, proportion), and time point for each outcome. Explanation of the clinical relevance of chosen efficacy and harm outcomes is strongly recommended	7-8 & 16-17
Participant timeline	#13	Time schedule of enrolment, interventions (including any run-ins and washouts), assessments, and visits for participants. A schematic diagram is highly recommended (see Figure)	12-15 & Figure 1
Sample size	#14	Estimated number of participants needed to achieve study objectives and how it was determined, including clinical and statistical assumptions supporting any sample size calculations	11
Recruitment	#15	Strategies for achieving adequate participant enrolment to reach target sample size	12

Methods:

Assignment of

For peer review only

**interventions (for controlled trials)**			
Allocation: sequence generation	#16a	Method of generating the allocation sequence (eg, computer-generated random numbers), and list of any factors for stratification. To reduce predictability of a random sequence, details of any planned restriction (eg, blocking) should be provided in a separate document that is unavailable to those who enrol participants or assign interventions	13
Allocation concealment mechanism	#16b	Mechanism of implementing the allocation sequence (eg, central telephone; sequentially numbered, opaque, sealed envelopes), describing any steps to conceal the sequence until interventions are assigned	13
Allocation: implementation	#16c	Who will generate the allocation sequence, who will enrol participants, and who will assign participants to interventions	12-13
Blinding (masking)	#17a	Who will be blinded after assignment to interventions (eg, trial participants, care providers, outcome assessors, data analysts), and how	N/A – Open label trial

For peer review only

Blinding (masking): emergency unblinding	#17b	If blinded, circumstances under which unblinding is permissible, and procedure for revealing a participant's allocated intervention during the trial	N/A – Open label trial
**Methods: Data collection, management, and analysis**			
Data collection plan	#18a	Plans for assessment and collection of outcome, baseline, and other trial data, including any related processes to promote data quality (eg, duplicate measurements, training of assessors) and a description of study instruments (eg, questionnaires, laboratory tests) along with their reliability and validity, if known. Reference to where data collection forms can be found, if not in the protocol	12-13 & 15-16
Data collection plan: retention	#18b	Plans to promote participant retention and complete follow-up, including list of any outcome data to be collected for participants who discontinue or deviate from intervention protocols	14

For peer review only

Data management	#19	Plans for data entry, coding, security, and storage, including any related processes to promote data quality (eg, double data entry; range checks for data values). Reference to where details of data management procedures can be found, if not in the protocol	15-16
Statistics: outcomes	#20a	Statistical methods for analysing primary and secondary outcomes. Reference to where other details of the statistical analysis plan can be found, if not in the protocol	16-17
Statistics: additional analyses	#20b	Methods for any additional analyses (eg, subgroup and adjusted analyses)	17
Statistics: analysis population and missing data	#20c	Definition of analysis population relating to protocol non-adherence (eg, as randomised analysis), and any statistical methods to handle missing data (eg, multiple imputation)	16
**Methods: Monitoring**			
Data monitoring: formal committee	#21a	Composition of data monitoring committee (DMC); summary of its role and reporting structure; statement of whether it is independent from the sponsor and competing interests; and reference to where further details about its charter can be found, if not in the	18

For peer review only

		protocol. Alternatively, an explanation of why a DMC is not needed	
Data monitoring: interim analysis	#21b	Description of any interim analyses and stopping guidelines, including who will have access to these interim results and make the final decision to terminate the trial	16
Harms	#22	Plans for collecting, assessing, reporting, and managing solicited and spontaneously reported adverse events and other unintended effects of trial interventions or trial conduct	15
Auditing	#23	Frequency and procedures for auditing trial conduct, if any, and whether the process will be independent from investigators and the sponsor	18
**Ethics and dissemination**			
Research ethics approval	#24	Plans for seeking research ethics committee / institutional review board (REC / IRB) approval	19
Protocol amendments	#25	Plans for communicating important protocol modifications (eg, changes to eligibility criteria, outcomes, analyses) to relevant parties (eg, investigators, REC / IRBs, trial participants, trial registries, journals, regulators)	18

For peer review only

Consent or assent	#26a	Who will obtain informed consent or assent from potential trial participants or authorised surrogates, and how (see Item 32)	12
Consent or assent: ancillary studies	#26b	Additional consent provisions for collection and use of participant data and biological specimens in ancillary studies, if applicable	13
Confidentiality	#27	How personal information about potential and enrolled participants will be collected, shared, and maintained in order to protect confidentiality before, during, and after the trial	19
Declaration of interests	#28	Financial and other competing interests for principal investigators for the overall trial and each study site	20-21
Data access	#29	Statement of who will have access to the final trial dataset, and disclosure of contractual agreements that limit such access for investigators	19
Ancillary and post trial care	#30	Provisions, if any, for ancillary and post-trial care, and for compensation to those who suffer harm from trial participation	N/A – no special compensation arrangements beyond rights as an NHS patient.

For peer review only

Dissemination policy: trial results	#31a	Plans for investigators and sponsor to communicate trial results to participants, healthcare professionals, the public, and other relevant groups (eg, via publication, reporting in results databases, or other data sharing arrangements), including any publication restrictions	19
Dissemination policy: authorship	#31b	Authorship eligibility guidelines and any intended use of professional writers	19
Dissemination policy: reproducible research	#31c	Plans, if any, for granting public access to the full protocol, participant-level dataset, and statistical code	N/A – unknown at this stage

Appendices

Informed consent materials	#32	Model consent form and other related documentation given to participants and authorised surrogates	Not submitted
Biological specimens	#33	Plans for collection, laboratory evaluation, and storage of biological specimens for genetic or molecular analysis in the current trial and for future use in ancillary studies, if applicable	13, 15

None The SPIRIT checklist is distributed under the terms of the Creative Commons Attribution License CC-BY-ND 3.0. This checklist can be completed online using https://www.goodreports.org/, a tool made by the EQUATOR Network in collaboration with Penelope.ai

For peer review only

BMJ Open

The NET-02 trial protocol: A multi-centre, randomised, parallel group, open-label, phase II, single-stage selection trial of liposomal irinotecan (nal-IRI) and 5-fluorouracil (5-FU)/folinic acid or docetaxel as second-line therapy in patients with progressive poorly differentiated extra-pulmonary neuroendocrine carcinoma (NEC)

Journal:	*BMJ Open*
Manuscript ID	bmjopen-2019-034527.R1
Article Type:	Protocol
Date Submitted by the Author:	17-Dec-2019
Complete List of Authors:	Craig, Zoe; University of Leeds, Clinical Trials Research Unit, Leeds Institute of Clinical Trials Research<br>Swain, Jayne; University of Leeds, Clinical Trials Research Unit, Leeds Institute of Clinical Trials Research<br>Batman, Emma; University of Leeds, Clinical Trials Research Unit, Leeds Institute of Clinical Trials Research<br>Wadsley, Jonathan; Weston Park Hospital<br>Reed, Nicholas; Beatson West of Scotland Cancer Centre<br>Faluyi, Olusola; Clatterbridge Cancer Centre NHS Foundation Trust<br>Cave, Judith; University Hospital Southampton NHS Foundation Trust, Department of Oncology<br>Sharma, Rohini; Imperial College London<br>Chau, Ian; Royal Marsden Hospital NHS Trust<br>Wall, Lucy; Western General Hospital<br>Lamarca, Angela; The Christie NHS Foundation Trust, Department of Medical Oncology; The University of Manchester, Division of Cancer Sciences<br>Hubner, R; The Christie NHS Foundation Trust, Department of Medical Oncology; The University of Manchester, Division of Cancer Sciences<br>Mansoor, Wasat; The Christie NHS Foundation Trust, Department of Medical Oncology<br>Sarker, Debashis; King's College Hospital<br>Meyer, Tim; University College London Cancer Institute<br>Cairns, David; University of Leeds, Clinical Trials Research Unit, Leeds Institute of Clinical Trials Research<br>Howard, Helen; University of Leeds, Clinical Trials Research Unit, Leeds Institute of Clinical Trials Research<br>Valle, Juan; The Christie NHS Foundation Trust, Department of Medical Oncology; The University of Manchester, Division of Cancer Sciences<br>McNamara, Mairéad ; The Christie NHS Foundation Trust, Department of Medical Oncology; The University of Manchester, Division of Cancer Sciences
**Primary Subject Heading**:	Oncology

Secondary Subject Heading:	Neurology, Diabetes and endocrinology
Keywords:	Neuroendocrine carcinoma, Randomised, Single-stage, Liposomal irinotecan, Docetaxel

SCHOLARONE™ Manuscripts

I, the Submitting Author has the right to grant and does grant on behalf of all authors of the Work (as defined in the below author licence), an exclusive licence and/or a non-exclusive licence for contributions from authors who are: i) UK Crown employees; ii) where BMJ has agreed a CC-BY licence shall apply, and/or iii) in accordance with the terms applicable for US Federal Government officers or employees acting as part of their official duties; on a worldwide, perpetual, irrevocable, royalty-free basis to BMJ Publishing Group Ltd ("BMJ") its licensees and where the relevant Journal is co-owned by BMJ to the co-owners of the Journal, to publish the Work in this journal and any other BMJ products and to exploit all rights, as set out in our licence.

The Submitting Author accepts and understands that any supply made under these terms is made by BMJ to the Submitting Author unless you are acting as an employee on behalf of your employer or a postgraduate student of an affiliated institution which is paying any applicable article publishing charge ("APC") for Open Access articles. Where the Submitting Author wishes to make the Work available on an Open Access basis (and intends to pay the relevant APC), the terms of reuse of such Open Access shall be governed by a Creative Commons licence – details of these licences and which Creative Commons licence will apply to this Work are set out in our licence referred to above.

Other than as permitted in any relevant BMJ Author's Self Archiving Policies, I confirm this Work has not been accepted for publication elsewhere, is not being considered for publication elsewhere and does not duplicate material already published. I confirm all authors consent to publication of this Work and authorise the granting of this licence.

For peer review only

The NET-02 trial protocol: A multi-centre, randomised, parallel group, open-label, phase II, single-stage selection trial of liposomal irinotecan (nal-IRI) and 5-fluorouracil (5-FU)/folinic acid or docetaxel as second-line therapy in patients with progressive poorly differentiated extra-pulmonary neuroendocrine carcinoma (NEC)

Authors: Craig Zoe[1], Swain Jayne[1], Batman Emma[1], Wadsley Jonathan[2], Reed Nicholas[3], Faluyi Olusola[4], Cave Judith[5], Sharma Rohini[6], Chau Ian[7], Wall Lucy[8], Lamarca Angela[9,10], Hubner Richard A[9,10], Mansoor Wasat[9], Sarker Debashis[11], Meyer Tim[12], Cairns David A[1], Howard Helen[1], Valle Juan W[9,10], McNamara Mairéad G[9,10*]

[1]*Clinical Trials Research Unit, Leeds Institute of Clinical Trials Research, University of Leeds, Leeds, United Kingdom*

[2]*Weston Park Hospital, Sheffield, United Kingdom*

[3]*Beatson Oncology Centre, Glasgow, United Kingdom*

[4]*Clatterbridge Cancer Centre, Wirral, United Kingdom*

[5]*University Hospital Southampton, Southampton, United Kingdom*

[6]*Imperial College, London, United Kingdom*

[7]*Royal Marsden, London, United Kingdom*

[8]*Western General Hospital, Edinburgh, United Kingdom*

[9]*Department of Medical Oncology, The Christie NHS Foundation Trust, Manchester, United Kingdom*

[10]*Division of Cancer Sciences, University of Manchester, Manchester, United Kingdom*

[11]*King's College Hospital, London, United Kingdom*

[12]*University College London Cancer Institute, London*, United Kingdom

* *Corresponding author:* Mairead.McNamara@christie.nhs.uk

Department of Medical Oncology,

The Christie NHS Foundation Trust/University of Manchester,

Wilmslow Road,

Withington,

Manchester M20 4BX,

United Kingdom

0161 446 8106

Word count: 4,071

For peer review only - http://bmjopen.bmj.com/site/about/guidelines.xhtml

ABSTRACT

Introduction: Poorly differentiated (PD), extra-pulmonary (EP), neuroendocrine carcinomas (NECs) are rare but aggressive neuroendocrine neoplasms (NENs). First-line treatment for advanced disease is an etoposide-platinum-based chemotherapy combination. There is no established second-line treatment for patients with PD-EP-NEC, and this is an area of unmet need.

Methods and analysis: NET-02 is a UK, multi-centre, randomised (1:1), parallel group, open-label, phase II, single-stage selection trial of liposomal irinotecan (nal-IRI)/5-fluorouracil (5-FU)/folinic acid or docetaxel as second-line therapy in patients with progressive PD-EP-NEC. One hundred and two eligible participants will be randomised to receive either nal-IRI/5-FU/folinic acid or docetaxel. The primary objective is to determine the 6-month progression-free survival (PFS) rate. The secondary objectives of this study are to determine PFS, overall survival, objective response rate, toxicity, quality of life and whether neuron-specific enolase is predictive of treatment response. If either treatment is found to have a 6-month PFS rate of at least 25%, that treatment will be considered for a phase III trial. If both treatments meet this target, pre-specified selection criteria will be applied to establish which treatment to take forward.

Ethics and dissemination: This study has ethical approval from the Greater Manchester Central Research Ethics Committee (Reference No. 18/NW/0031) and clinical trial authorisation from the Medicine and Healthcare Products Regulatory Agency. Results will be published in peer-reviewed journals and uploaded to the EU Clinical Trials Register.

Trial registration: ISRCTN10996604, ClinicalTrials.gov: NCT03837977, EudraCT Number: 2017-002453-11

Key words: Neuroendocrine carcinoma, randomised, single-stage, liposomal irinotecan, docetaxel

For peer review only

For peer review only - http://bmjopen.bmj.com/site/about/guidelines.xhtml

ARTICLE SUMMARY

Strengths and limitations of this study

- The trial is designed to ensure, with a high probability, that the most efficacious treatment is selected to be taken forward to a phase III trial.
- Prospectively defined decision criteria in this trial will enable earlier planning of a phase III trial if these targets are reached.
- The trial is not powered to directly compare the two treatment arms in this study.

For peer review only

For peer review only - http://bmjopen.bmj.com/site/about/guidelines.xhtml

INTRODUCTION

Neuroendocrine carcinomas

Neuroendocrine carcinomas (NECs) are a rare, high-grade, poorly differentiated (PD) form of neuroendocrine neoplasms (NENs).[1] The annual incidence of PD extra-pulmonary (EP) NEC is approximately one diagnosis per 100,000 persons.[2 3] These tumours are characterised by aggressive histological features; high Ki-67 index (>20% by definition, but usually higher (>75%)),[4] extensive necrosis and nuclear atypia, and are classified as NEC grade 3 according to the World Health Organisation (WHO) 2010 classification.[5]

First-line treatment for PD-EP-NECs has remained largely unchanged since a study in the early 1990s reported anti-tumour activity and high tumour response rates (RRs) produced by an etoposide-platinum combination.[6] Nevertheless, disease progression invariably occurs in patients during or following completion of first-line therapy, and a standard second-line treatment is yet to be determined.

Current second-line treatment options for patients with a NEC diagnosis

For patients with advanced PD-EP-NEC, combination regimens such as irinotecan, 5-fluorouracil (5-FU) and folinic acid are a second-line treatment option currently used, without robust trial evidence.[7] This combination has been recommended for patients with a NEC diagnosis with a Ki-67 ≥55%, whereas some literature recommends temozolomide based-combinations for those with a Ki-67 <55%.[8 9] In devising treatment strategies for PD-EP-NEC, many refer to the extensive literature on high-grade NEC of the lung, for which docetaxel is a second-line therapy option.[9]

Several small retrospective studies have published results for the outcomes of second-line chemotherapy after failure of the etoposide-platinum combination in patients with grade 3 NECs.[7-13] The NORDIC-NEC study reported predictive and prognostic factors for treatment and survival in 305

For peer review only - http://bmjopen.bmj.com/site/about/guidelines.xhtml

patients with advanced gastrointestinal NEC.[9] Second-line chemotherapy was administered to 100 patients; of these, 35 received temozolomide-based chemotherapy and 20 received docetaxel-based chemotherapy. Of 84 evaluable patients, the RR was 18%. Those whose tumours had a Ki-67 <55% had a lower RR, but better survival than patients whose tumours had a Ki-67 ≥55%. The median overall survival (OS) for patients treated with first-line platinum-based chemotherapy in the advanced setting is 11-16.4 months.[9 14] In a systematic review and meta-analysis of second-line treatment in 595 patients with advanced PD-EP-NEC, the median RR was 18%, the median progression-free survival (PFS) was 2.5 months (range 1.2-6.0) and the median OS was 7.6 months (range 3.2-22).[15]

Liposomal irinotecan (nal-IRI)

Irinotecan, a topoisomerase I inhibitor, works to arrest uncontrolled cell growth by preventing the unwinding of deoxyribonucleic acid (DNA), therefore preventing cell replication and tumour growth.[16]

Liposomal irinotecan (nal-IRI) (ONIVYDE®, Servier) is irinotecan encapsulated in a liposome drug delivery system. This stable liposome formulation of irinotecan has several attributes that may provide an improved therapeutic index; controlled and sustained release, high intravascular drug retention and enhanced permeability.[16 17] The improved pharmacokinetics and bio-distribution of nal-IRI in comparison to irinotecan may have clinical benefit in patients with NEC.

Pharmacokinetic studies have demonstrated that once irinotecan is released from the liposomes, the conversion to the active metabolite, SN-38, is similar to that of un-encapsulated irinotecan.[16 18] Thus, nal-IRI and un-encapsulated irinotecan have demonstrated similar adverse reactions (ARs) in patients, the most common of which include gastrointestinal events and myelosuppression.[16 18]

For peer review only - http://bmjopen.bmj.com/site/about/guidelines.xhtml

Rationale for the use of nal-IRI in combination with 5-FU and folinic acid

Pre-clinical evidence supports the hypothesis that nal-IRI modifies the tumour microenvironment in a manner that should make tumours more susceptible to 5-FU/folinic acid, through decreasing tumour hypoxia and increasing small molecule perfusion.[19 20]

Given the relative absence of overlapping toxic effects among nal-IRI, 5-FU and folinic acid, a regimen combining these agents was studied in a phase I, dose-escalation trial of solid tumours.[21] Among the 15 efficacy-evaluable participants, the overall disease control rate was 73.3%. Among the six participants who received the nal-IRI maximum tolerated dose of 80 mg/m^2, the objective response rate (ORR) and disease control rate were 16.7% and 83.3%, respectively.

In the NAPOLI-1 phase III trial of nal-IRI, with or without 5-FU and folinic acid, versus 5-FU and folinic acid alone, in the treatment of patients with metastatic pancreatic ductal adenocarcinoma after receiving gemcitabine-based therapy, an increase in OS for those treated with a combination of nal-IRI and 5-FU/folinic acid was reported compared to those treated with 5-FU and folinic acid alone (hazard ratio for survival (HR) 0.67, 95% confidence interval (CI) 0.49-0.92).[22]

Rationale for the use of docetaxel

The National Comprehensive Cancer Network Clinical Practice Guidelines in Oncology (NCCN Guidelines®), for the treatment of small cell and non-small cell lung cancer, include docetaxel as a second-line treatment option in patients who have progressed on primary etoposide-platinum combination therapy.[23 24] Based on observed RRs, survival, quality of life (QoL) and toxicities, the optimal dose of docetaxel in pre-treated patients with non-small cell lung cancer is 75mg/m^2 every 3 weeks.[25]

For peer review only - http://bmjopen.bmj.com/site/about/guidelines.xhtml

Study rationale and aim

Treatment of patients with advanced PD-EP-NEC, to date, has been analogous to that of high-grade NEC (small cell or non-small cell cancer) of the lung.[6] The standard arm of NET-02 is that used in high-grade lung NEC, of which docetaxel is a second-line therapy option,[23] and combination regimens such as irinotecan/5-FU are a second-line therapy option currently used, without trial evidence, for this subset of patients.[7] Prospective collaborative trials, with translational end-points, are warranted and may inform future biomarker-driven studies.

Therefore, the overall aim of this trial is to assess the efficacy of nal-IRI/5-FU/folinic acid or docetaxel, separately, as second-line therapy in patients with progressive PD-EP-NEC, with selection criteria applied to establish which treatment to take forward into a phase III trial.

METHODS AND ANALYSIS

Trial objectives

The primary objective of the trial is to determine the 6-month PFS rate, defined as a binary outcome (progression-free or not) within the timeframe of treatment start date until 6 months post-randomisation.

The secondary objectives of the trial are to determine:

- Progression-free survival (defined as the time from randomisation to progression or death from any cause)
- Overall survival (defined as the time from randomisation to death from any cause)
- Objective response rate at 6 months post-randomisation (defined using the Response Evaluation Criteria in Solid Tumours (RECIST) v1.1 measurements)[26]
- Toxicity as per Common Terminology Criteria for Adverse Events (CTCAE) version 5.0
- Quality of life (defined using European Organisation for Research and Treatment of Cancer

For peer review only - http://bmjopen.bmj.com/site/about/guidelines.xhtml

(EORTC) quality of life validated questionnaires (QLQ) C30 (EORTC QLQ-C30)[27] and GINET21 (EORTC QLQ-GINET21))[27 28]

- Association between neuron-specific enolase concentration and treatment response

Additional exploratory objectives, analysing participant samples, will include:

- Quantification of circulating tumour cells (CTCs) and circulating tumour DNA at baseline, 6 weeks and on progression, to identify any correlation with disease-related outcomes
- Molecular profiling of CTCs, circulating tumour DNA and tumour tissue (further immunohistochemistry on tumour tissue may also be required) to identify any correlation with disease-related outcomes
- Generation of mouse models of PD-EP-NEC

Trial design

The NET-02 trial is a United Kingdom (UK), multi-centre, randomised (1:1), parallel group, open-label, phase II, single-stage selection trial of nal-IRI/5-FU/folinic acid or docetaxel as second-line therapy in patients with progressive PD-EP-NEC.

The design is an adaptation of a one-stage trial design proposed by Simon, Wittes and Ellenberg, where the A'Hern design is first implemented to assess efficacy of each treatment separately, to ensure a pre-specified minimum level of activity prior to selection.[29] Should both treatments be sufficiently efficacious, pre-specified selection criteria are then applied to establish which treatment to take forward into a phase III trial. The intention of the trial is to show that the regimens are sufficiently active in this patient population, but not to show that one regimen is significantly superior to the other.

For peer review only - http://bmjopen.bmj.com/site/about/guidelines.xhtml

The A'Hern method is advantageous over other single-stage designs, since it uses the exact binomial distribution, as opposed to a normal approximation to the binomial distribution which can lead to substantial error in small trials.[30] Additionally, prospectively defined decision criteria, specified below, are applied, which if reached, could enable earlier planning for a phase III follow-on trial.

Participants will be randomised to receive either nal-IRI/5-FU/folinic acid, administered every 14 days, or docetaxel, administered every 21 days. Trial treatment will continue until progressive disease, intolerable toxicity, delay of treatment for more than 28 days, development of any condition or occurrence of any event, which, in the opinion of the local investigator, justifies discontinuation of treatment, participant request or until 6 months after the last participant is randomised, whichever occurs first. Figure 1 displays the full trial schema.

Trial population and sample size

The NET-02 trial will recruit patients diagnosed with PD-EP-NEC (Ki-67 >20% and grade 3, confirmed by histology). Patients will be eligible for the trial if they meet all of the inclusion criteria and do not satisfy any of the exclusion criteria listed in Table 1.

Table 1 | NET-02 inclusion and exclusion criteria

Inclusion criteria	Exclusion criteria
1. Age ≥18 years and life expectancy >3 months.<br>2. Diagnosed with poorly differentiated (as defined by the WHO in 2010, Ki-67 >20%) EP-NEC (grade 3, confirmed by histology). Carcinoma of unknown primary is allowed if lung primary has been excluded following review by the multi-disciplinary team.<br>3. Prior treatment with first-line platinum-based chemotherapy for NEC in the advanced setting and ≥28 days from Day 1 of the previous treatment cycle.<br>4. Documented radiological evidence of disease progression OR discontinuation of first-line platinum-based chemotherapy due to intolerance.<br>5. Measurable disease according to RECIST 1.1.<br>6. Eastern Co-operative Oncology Group (ECOG) performance status ≤2.	1. Known or suspected allergy or hypersensitivity reaction to any of the components of study treatment or their excipients.<br>2. Use (including self-medication) within one week of randomisation and for the duration of the study of any of the following: St. John's wort, grapefruit, Seville oranges, medicines known to inhibit UGT1A1 (e.g. atazanavir, gemfibrozil, indinavir) and medicines known to inhibit or induce either CYP3A4 or CYP3A5.<br>3. Previous treatment (for NEC) with any of the components of combination chemotherapy regimens detailed in this study (nal-IRI, 5-FU, irinotecan, topoisomerase inhibitors or taxane-based therapy).<br>4. Incomplete recovery from previous therapy in the opinion of the investigator (surgery/adjuvant therapy/radiotherapy/chemotherapy in

For peer review only - http://bmjopen.bmj.com/site/about/guidelines.xhtml

Inclusion criteria	Exclusion criteria
7. Adequate renal function with serum creatinine ≤1.5 times upper limit of normal (ULN) and creatinine clearance ≥30ml/min according to Cockroft-Gault or Wright formula. If the calculated creatinine clearance is <30ml/min, glomerular filtration rate (GFR) may be assessed using either Cr51-EDTA or 99mTc-DTPA clearance method to confirm if GFR is ≥30 ml/min).<br>8. Adequate haematological function: Hb ≥90g/L, WBC ≥3.0 x 10^9/L, ANC ≥1.5 x 10^9/L, platelet count ≥100 x 10^9/L.<br>9. Adequate liver function: serum total bilirubin ≤1.5 x ULN (biliary drainage is allowed for biliary obstruction) and ALT and/or AST ≤2.5 x ULN in the absence of liver metastases, or ≤5 x ULN in the presence of liver metastases.<br>10. A negative pregnancy test is required at registration in women of childbearing potential.<br>11. Men and women of reproductive potential must agree to use a highly effective form of contraception during the study and for 6 months following the last dose of trial treatment. In addition, male participants should use a condom during study participation and for 6 months following the last dose of trial treatment.<br>12. Patients must be able to provide written informed consent.<br>13. Patients must be able and willing to comply with the terms of the protocol.	advanced setting), including ongoing peripheral neuropathy of > Common Terminology Criteria for Adverse Events (CTCAE) v5.0 grade 2 from previous platinum-based therapy.<br>5. Concurrent palliative radiotherapy involving target lesions used for this study (<28 days from discontinuation of radiotherapy). Radiotherapy for non-target lesions is allowed if other target lesions are available outside the involved field.<br>6. Patients must not have a history of other malignant diseases (within the previous 3 years, and there must be no evidence of recurrence), other than:<br>• EP-NEC.<br>• Non-melanoma skin cancer where treatment consisted of resection only or radiotherapy.<br>• Ductal carcinoma *in situ* (DCIS) where treatment consisted of resection only.<br>• Cervical carcinoma *in situ* where treatment consisted of resection only.<br>• Superficial bladder carcinoma where treatment consisted of resection only.<br>7. Documented brain metastases, unless adequately treated (surgery or radiotherapy only), with no evidence of progression and neurologically stable off anticonvulsants and steroids.<br>8. Clinically significant gastrointestinal disorder (in the opinion of the treating clinician), including hepatic disorders, bleeding, inflammation, obstruction, or diarrhoea > CTCAE grade 1 (at time of study entry).<br>9. Severe arterial thromboembolic events (myocardial infarction, unstable angina pectoris, stroke) less than 6 months before inclusion.<br>10. New York Heart Association (NYHA) Class III or IV congestive heart failure, ventricular arrhythmias or uncontrolled blood pressure.<br>11. Severe bone marrow failure or bone marrow depression after radiotherapy or treatment with other antineoplastic agents (defined as haematological values of haemoglobin or white blood cells or neutrophils or platelets not meeting inclusion criteria).<br>12. Known active hepatitis B virus, hepatitis C virus or HIV infection.<br>13. Active chronic inflammatory bowel disease.<br>14. Breastfeeding women.<br>15. Evidence of severe or uncontrolled systemic diseases, which, in the view of the treating clinician, makes it undesirable for the patient to participate in the trial.<br>16. Evidence of significant clinical disorder or laboratory finding which, in the opinion of the

For peer review only - http://bmjopen.bmj.com/site/about/guidelines.xhtml

Inclusion criteria	Exclusion criteria
	treating clinician, makes it undesirable for the patient to participate in the trial.<br>17. Medical or psychiatric conditions that impair the ability to give informed consent.<br>18. Any other serious uncontrolled medical conditions (in the opinion of the treating clinician).

One hundred and two eligible participants will be randomised to receive either nal-IRI/5-FU/folinic acid or docetaxel. Allowing for a 5% drop-out rate, this will provide 80% power for demonstrating that the one-sided 95% CI for the 6-month PFS rate excludes 15%, if the true rate is at least 30%, where 30% is the required level of efficacy and a rate of 15% or less would give grounds for rejection, i.e. the relevant treatment would be considered not to have reached an acceptable level of efficacy to warrant further evaluation. The proportions of 15% and 30% were chosen in line with existing literature; of those who reported the proportion progression-free at 6 months, the lowest was approximately 15%[11] and the highest approximately 25%.[7] Therefore, for either trial treatment to be taken forward for further research, they should provide estimates that are at least as good as the lower value and aim to improve on the higher value.

A treatment arm may be considered for further evaluation using the treatment selection process described below, if at least 12 out of 48 evaluable participants are progression-free at 6 months (equating to a success rate of 25%, with a lower one-sided 95% confidence limit of 15.1%).

Treatment selection

If both treatments successfully exceed the pre-defined criteria, having lower one-sided 95% confidence limits greater than 15%, Simon's design proposes that the treatment with the higher PFS rate at 6 months should be selected, regardless how small its advantage over the other treatment appears.[29] Nevertheless, to ensure that the more efficacious treatment is selected with a high probability, if the difference in the 6-month PFS rates is less than 5%, additional selection criteria,

For peer review only - http://bmjopen.bmj.com/site/about/guidelines.xhtml

including toxicity rates and QoL score, will be considered. If only one of the treatments successfully exceeds the pre-defined criteria, this treatment will be selected for further investigation.

Recruitment, registration and randomisation

Participants will be recruited from 16 UK sites (see supplementary material) over a 37-month period. Potential participants will be approached, regarding trial participation, during the standard clinic visit at which their progression following first-line chemotherapy is discussed and will be provided with a verbal and written explanation of the trial. Patients, who provide written informed consent, to the site Principal Investigator or delegate, will be registered onto the trial. Consent to the use of blood samples for future projects and mouse model generation (The Christie NHS Foundation Trust participants only) is optional.

Recruitment of participants to the NET-02 trial requires trial-specific investigations to confirm eligibility. Consequently, recruitment is a two-step process involving registration and randomisation.

Initial registration will involve all patients who have provided written informed consent. Patients will undergo investigations to confirm eligibility including a full blood count, biochemistry and renal function assessment, an electrocardiogram (ECG) and a pregnancy test (if applicable) to confirm that they satisfy the eligibility criteria specified in Table 1.

Once all other screening investigations are successfully completed and prior to meeting with the clinician and randomisation, two baseline QoL questionnaires (EORTC QLQ-C30[27] and EORTC QLQ-GINET21[28]) will be completed.

Patients identified as eligible, following the eligibility assessments, will be randomised. If more than 14 days have elapsed since the initial eligibility blood tests, these must be repeated prior to randomisation, to ensure that the patient remains eligible. Registration and randomisation will be

For peer review only - http://bmjopen.bmj.com/site/about/guidelines.xhtml

performed centrally using either the Leeds Clinical Trials Research Unit (CTRU) automated telephone or web-based system.

A minimisation program, which incorporates a random element, will be used for randomisation to ensure treatment groups are well balanced for the following characteristics:

- Hospital site
- Ki-67 marker (<55%, ≥55%)
- Eastern Co-operative Oncology Group (ECOG) performance status (0/1, 2),
- Presence of liver metastases (yes, no)
- Response to first-line platinum-based chemotherapy (resistant disease (progression ≤6 months from completion of platinum-based therapy), sensitive disease (progression >6 months from completion of platinum-based therapy), platinum intolerant).

Following randomisation, baseline assessments will be conducted. These will include; medical history, demographics, baseline symptoms, physical examination, vital signs, computed tomography (CT) scan (or magnetic resonance imaging (MRI) scan, if appropriate) of the thorax-abdomen-pelvis and staging within 28 days of starting trial treatment, one 10ml blood sample for local measurement of neuron-specific enolase and two 10ml blood samples for central translational research. Confirmation of availability of archival paraffin-embedded tissue for translational research will also be sought. An additional 10ml blood sample may be taken for mouse model development for consenting participants from The Christie NHS Foundation Trust.

Interventions

Nal-IRI (ONIVYDE®, Servier), folinic acid and 5-FU will be administered sequentially. The recommended dose and regimen of nal-IRI is 80 mg/m^2 body surface area (BSA) intravenously over 90 minutes (±10

For peer review only - http://bmjopen.bmj.com/site/about/guidelines.xhtml

minutes), followed by folinic acid as per local standard practice (recommended dose is 350 mg fixed dose), followed by 5-FU 2400 mg/m^2 BSA intravenously over 46 hours. Following cycle 1, subsequent doses will be administered every 14 days (+3 days/-1 day). Where it is not possible to administer nal-IRI due to toxicity, 5-FU/folinic acid can be administered as a monotherapy.

Docetaxel will be administered at a dose of 75 mg/m^2 BSA as an intravenous (IV) infusion over 60 minutes, or as per local standard practice. Following cycle 1, subsequent doses will be administered every 21 days (+3 days/-1 day).

Dosing may be postponed for up to 28 days from when it was due, to allow for (but not limited to) recovery from treatment-related toxicities, infection, or following patient request. In the event of a delay due to toxicity, a dose modification (see supplementary material) may be required at subsequent cycles following a dose delay. If a patient's dose is reduced due to toxicity, it will remain reduced for the duration of treatment. Patients who have already received two dose reductions and experience additional toxicities that would require further dose reduction should discontinue study medication. However, in the event that the participant is deriving clinical benefit and the treating clinician would prefer to continue treatment, an additional dose reduction may be permitted at the discretion of the Chief Investigator or delegate. If the toxicity recovery duration (to ≤Grade 2 CTCAE v5.0 or baseline) is more than 28 days, the participant should discontinue trial treatment. Participants who have prematurely discontinued treatment will continue to attend 8-weekly clinic visits for CT scans and have follow-up data collected, unless the participant withdraws consent for follow-up visits and further data collection.

All concurrent medical conditions and complications of the underlying malignancy will be treated at the discretion of the treating physician according to local standards of medical care. Participants can receive analgesics, antiemetics, antibiotics, antipyretics, and blood products as necessary. However, the use of warfarin-type anticoagulant therapies is not permitted.

For peer review only

Treatment cycle assessments

Participants on the nal-IRI/5-FU/folinic acid treatment arm will have 2-weekly treatment cycles. Participants on the docetaxel treatment arm will have 3-weekly treatment cycles.

Assessments carried out on the first day of each treatment cycle will include; laboratory assessments, clinical evaluation, vital signs, ECOG performance status, physical examination, details of concomitant medication and toxicity assessment (from cycle 2 onwards). Translational research blood samples and QoL questionnaires will be collected at 6-weekly intervals and at disease progression. A CT or MRI scan will be carried out 8-weekly (±7 days) from treatment start until disease progression or until 6 months after the last participant is randomised, whichever occurs first. Disease progression will be defined as the date of the CT or MRI scan that identifies disease progression. In the rare circumstances that disease progression is determined clinically and it is not appropriate to confirm it radiologically, the date of progression will be defined as the date of documented clinical disease progression.

Safety

Adverse events (AEs) and adverse reactions (ARs) will be collected on the first day of each treatment cycle from cycle 2 onwards. Serious adverse events (SAEs), serious adverse reactions (SARs) and suspected unexpected serious adverse reactions (SUSARs) will be collected from registration. All AEs, ARs and SAEs will be collected until 28 days after the last dose of trial treatment was administered; SARs and SUSARs will be collected until the end of the study.

Data collection

Data will be collected using paper case report forms (CRFs) and entered into a validated trial database by the CTRU, where data quality will be monitored. Automatic and manual validation of entered data will be conducted. Data items relating to the safety and rights of individual participants will be dealt

For peer review only - http://bmjopen.bmj.com/site/about/guidelines.xhtml

with as a priority. Data items required for the primary endpoint analysis will be manually checked at the CTRU. Missing data will be chased until it is either received or confirmed as not available at the trial analysis stage.

Statistical analysis

A full statistical analysis plan (SAP) will be written before any analysis is undertaken.

The primary analysis population will be defined as those who have received at least one dose of the protocol treatment. Individuals will be analysed according to the treatment that they received rather than that which they were randomised to receive. The QoL population is defined as any individual who returned at least one QoL questionnaire. Unless otherwise stated, the analysis will be conducted separately for each treatment group as per the primary analysis population.

All analyses will use a 5% significance level. The primary endpoint will be presented with a 1-sided CI, whilst secondary endpoints will be presented with 2-sided CIs. No formal interim analyses are scheduled to occur; hence, no statistical testing will take place until final analysis, which will occur once all randomised participants have reached the primary endpoint. Nevertheless, the Data Monitoring and Ethics Committee (DMEC) will receive full reports, at least annually and safety reports at least 6-monthly, to monitor participant safety and trial progress, and they may prematurely terminate the trial if necessary.

Primary endpoint analysis of the proportion of participants progression-free at 6 months post-randomisation will be calculated using exact methods. If the one-sided CI for either treatment from this analysis includes 15%, then that treatment will not be considered for a phase III trial. An individual is defined to have achieved the primary endpoint if they do not progress within the timeframe of treatment start date until 6 months post-randomisation. If an individual dies or is lost to follow-up, without confirmation of disease progression, within 6-months post-randomisation, they will be

For peer review only - http://bmjopen.bmj.com/site/about/guidelines.xhtml

considered to have not achieved this endpoint and will be censored at the date of death or date last known to be alive and progression-free.

Secondary endpoint analysis will include summary statistics and Kaplan-Meier survival curves for PFS and OS, summaries of the number and cause of deaths, and calculation of the ORR (defined as the proportion of participants achieving at least a partial response (PR) within 6-months post randomisation).

Safety analyses will summarise AEs, ARs, SAEs, SARs, SUSARs, and pregnancies. Line listings of SAEs will be generated and will include details on expectedness, causality, relationship to the trial treatment and outcome.

Quality of life will be summarised using mean scores for each subscale and repeated measures models will be employed to investigate changes in health-related QoL over time for each treatment group, using the QoL population.

In the event that both treatment groups meet the specified threshold for the primary endpoint, and show a similar level of efficacy, toxicity and QoL data will inform which treatment to investigate in further research.

Summary statistics for the concentration of neuron-specific enolase at each time point will be estimated. The baseline concentration of neuron-specific enolase will be analysed to assess whether it is associated with response to treatment at 6 months post-randomisation, via an ordinal logistic regression model, adjusting for the stratification factors (excluding hospital site) and any appropriate interaction variables.

Exploratory analysis of the primary and selected secondary endpoints (PFS, OS and ORR) will be done using logistic or Cox regression, as appropriate. All models will be adjusted for the stratification factors (excluding hospital site). Subgroup analysis of the primary and selected secondary endpoints (as above) will include investigation of gender, age, Ki-67 value and morphology of NEC. All exploratory

For peer review only - http://bmjopen.bmj.com/site/about/guidelines.xhtml

For peer review only

and subgroup analyses will be considered as hypothesis-generating rather than as confirmatory if significant differences are found. Further exploratory and subgroup analyses beyond that described may be undertaken.

Trial monitoring

A Trial Monitoring Plan will be developed by the Trial Management Group (TMG) and agreed by the Trial Steering Committee (TSC), based on the trial risk assessment. The TMG, comprising the Chief Investigator, CTRU team, other key trial staff, a nursing representative and a patient and public involvement (PPI) representative will be assigned responsibility for the clinical setup, on-going management, promotion of the trial and the interpretation and publishing of the results. The TSC and DMEC will provide independent oversight of the study and will be responsible for monitoring the study conduct. The TSC, comprising a statistician, an oncologist and a PPI representative will provide overall supervision of the trial. The DMEC, comprised of two gastroenterologists, an oncologist (all with experience in the treatment of patients with NENs) and a statistician, will review the safety and ethics of the study alongside the trial progress, as overseen by the TSC. The DMEC will review confidential safety reports at least 6-monthly and the DMEC and TSC will meet separately, at least annually, to discuss trial progress.

Patient and public involvement

Patient and public involvement representatives are involved in the design and overall direction of the trial through their roles in the TMG and the TSC. As part of the TMG, the PPI representative has been involved in protocol development and the preparation of the patient information and informed consent trial documentation. As part of the TSC, the PPI representative will provide advice regarding trial design and conduct, and will be involved in monitoring trial progress and patient safety.

For peer review only - http://bmjopen.bmj.com/site/about/guidelines.xhtml

For peer review only

ETHICS AND DISSEMINATION

The NET-02 trial opened to recruitment on 16th November 2018. At the time of submission, 12 centres out of 16 are open to recruitment, and 17 participants have been randomised into the trial. The trial is currently adhering to version 3.0 of the protocol (approved 20th September 2018), with all sites opening to this version of the protocol. The trial is sponsored by The Christie NHS Foundation Trust, coordinated by Leeds CTRU and funded by Servier (unrestricted grant). The trial is registered on the International Standard Randomised Controlled Trial registry (ISRCTN: 10996604), European Clinical Trials Database (EudraCT: 2017-002453-11) and ClinicalTrials.gov: NCT03837977.

This study has ethical approval from the Greater Manchester Central Research Ethics Committee (Reference No. 18/NW/0031) and clinical trial authorisation from the Medicine and Healthcare Products Regulatory Agency. The trial will be conducted in accordance with Good Clinical Practice. Trial results will be published in peer-reviewed journals and will be reported in line with the Consolidated Standards of Reporting Trials (CONSORT) guidelines.[31] Authorship will be decided according to the International Committee of Medical Journal Editors (ICMJE) criteria for authorship.[32] All publications will be reviewed by the Sponsor and funder prior to publication. To maintain the scientific integrity of the trial, data will not be released prior to the first publication of the analysis of the primary endpoint, for either trial publication or oral presentation purposes, without the permission of the Sponsor and TSC. Research results will also be uploaded to the EU Clinical Trials Register.

All information collected during the course of the trial will be kept strictly confidential. Information will be held securely at the CTRU. The CTRU will comply with all aspects of the General Data Protection Regulation (GDPR) 2018.[33] The trial staff at the participating sites will be responsible for ensuring that any data or documentation sent to the CTRU is appropriately anonymised. At the end of the trial, data will be securely archived in line with the Sponsor's procedures for a minimum of 15 years. Data held

For peer review only - http://bmjopen.bmj.com/site/about/guidelines.xhtml

by the CTRU will be archived in the Sponsor archive facility, and site data and documents will be archived at the sites. Following authorisation from the Sponsor, arrangements for confidential destruction will then be made.

For peer review only

For peer review only - http://bmjopen.bmj.com/site/about/guidelines.xhtml

AUTHOR CONTRIBUTIONS:

Conception and design of the NET-02 trial: JS, DAC, HH, JWV and MMN. Development of the protocol and patient information sheet: JS, DAC, HH, DS, OF, TM, JWV and MMN. Writing of manuscript: ZC, JS, DAC, and MMN. Review of manuscript: EB, JW, NR, OF, JC, RS, IC, LW, AL, RAH, MW, DS, TM and HH.

All authors have read and approved the final manuscript.

ACKNOWLEDGEMENTS:

Thanks to Dr Alison Backen (Project Manager, The Christie NHS Foundation Trust) for her contribution to initial protocol development.

FUNDING:

This research is investigator-initiated and funded by an unrestricted educational grant from Servier (grant reference number 016-34263). This work was also supported by Core Clinical Trials Unit Infrastructure from Cancer Research UK (C7852/A25447).

SPONSOR: The Christie NHS Foundation Trust, Wilmslow Road, Manchester, M20 4BX, United Kingdom. Sponsor reference: CFTSp116

COMPETING INTERESTS:

JW reports grants and personal fees from AstraZeneca, grants and personal fees from Sanofi-Genzyme, personal fees and non-financial support from Celgene, personal fees from Eisai, personal

For peer review only

fees and non-financial support from Ipsen, personal fees and non-financial support from Novartis, non-financial support from Imaging Equipment Ltd, outside the submitted work.

IC reports advisory role for Eli-Lilly, Bristol Meyers Squibb, MSD, Bayer, Roche, Merck-Serono, Five Prime Therapeutics, AstraZeneca, Oncologie International, Pierre Fabre; research funding from Eli-Lilly, Janssen-Cilag, Sanofi Oncology, Merck-Serono; honorarium from Eli-Lilly.

AL received travel and educational support from Ipsen, Pfizer, Bayer, AAA, Sirtex Medical, Novartis, Mylan and Delcath Systems; speaker honoraria from Merck, Pfizer, Ipsen and Incyte; advisory honoraria from EISAI and Nutricia; she is a member of the Knowledge Network and NETConnect Initiatives funded by Ipsen.

DS reports personal fees from MSD, personal fees and non-financial support from EISAI, personal fees and non-financial support from Ipsen, personal fees from Bayer, non-financial support from Mina Therapeutics, personal fees from Pfizer, personal fees from Novartis, outside the submitted work.

TM reports grants from Bayer, grants from BTG, personal fees from BMS, personal fees from EISAI, personal fees from AstraZeneca, personal fees from Tarveda , personal fees from Ipsen, personal fees from MSD, outside the submitted work.

DAC reports grants and non-financial support from Servier, during the conduct of the study.

HH reports grants and non-financial support from Servier, during the conduct of the study.

JWV reports Consulting or Advisory role for Ipsen, Novartis, AstraZeneca, Merck, Delcath Systems, Agios, Pfizer, PCI Biotech, Incyte, Keocyt, QED, Pieris Pharmaceuticals, Genoscience Pharma, Mundipharma EDO; Honoraria from Ipsen; and Speakers' Bureau for Novartis, Ipsen, NuCana and Imaging Equipment Limited.

MMN has received research grant support from Servier, Ipsen and NuCana. She has received travel and accommodation support from Bayer and Ipsen and speaker honoraria from Pfizer, Ipsen and NuCana. She has served on advisory boards for Celgene, Ipsen, Sirtex and Baxalta.

For peer review only - http://bmjopen.bmj.com/site/about/guidelines.xhtml

EXCLUSIVE LICENCE:

I, the Submitting Author has the right to grant and does grant on behalf of all authors of the Work (as defined in the below author licence), an exclusive licence and/or a non-exclusive licence for contributions from authors who are: i) UK Crown employees; ii) where BMJ has agreed a CC-BY licence shall apply, and/or iii) in accordance with the terms applicable for US Federal Government officers or employees acting as part of their official duties; on a worldwide, perpetual, irrevocable, royalty-free basis to BMJ Publishing Group Ltd ("BMJ") its licensees and where the relevant Journal is co-owned by BMJ to the co-owners of the Journal, to publish the Work in BMJ Open and any other BMJ products and to exploit all rights, as set out in our licence.

The Submitting Author accepts and understands that any supply made under these terms is made by BMJ to the Submitting Author unless you are acting as an employee on behalf of your employer or a postgraduate student of an affiliated institution which is paying any applicable article publishing charge ("APC") for Open Access articles. Where the Submitting Author wishes to make the Work available on an Open Access basis (and intends to pay the relevant APC), the terms of reuse of such Open Access shall be governed by a Creative Commons licence – details of these licences and which Creative Commons licence will apply to this Work are set out in our licence referred to above.

FIGURE LEGEND: Figure 1| Trial Schema

For peer review only

REFERENCES

1. Leoncini E, Boffetta P, Shafir M, et al. Increased incidence trend of low-grade and high-grade neuroendocrine neoplasms. Endocrine 2017;**58**(2):368-79.
2. Dasari A, Mehta K, Byers LA, et al. Comparative study of lung and extrapulmonary poorly differentiated neuroendocrine carcinomas: A SEER database analysis of 162,983 cases. Cancer 2018;**124**(4):807-15.
3. Korse CM, Taal BG, van Velthuysen MLF, et al. Incidence and survival of neuroendocrine tumours in the Netherlands according to histological grade: Experience of two decades of cancer registry. European Journal of Cancer 2013;**49**(8):1975-83.
4. Brenner B, Tang LH, Shia J, et al. Small Cell Carcinomas of the Gastrointestinal Tract: Clinicopathological Features and Treatment Approach. Seminars in Oncology 2007;**34**(1):43-50.
5. Bosman FT, Carneiro F, Hruban RH, et al. WHO classification of tumours of the digestive system, 4th edition. WHO Press 2010.
6. Moertel CG, Kvols LK, O'Connell MJ, et al. Treatment of neuroendocrine carcinomas with combined etoposide and cisplatin. Evidence of major therapeutic activity in the anaplastic variants of these neoplasms. Cancer 1991;**68**(2):227-32.
7. Hentic O, Hammel P, Couvelard A, et al. FOLFIRI regimen: an effective second-line chemotherapy after failure of etoposide-platinum combination in patients with neuroendocrine carcinomas grade 3. Endocrine Related Cancer 2012;**19**(6):751-57.
8. Welin S, Sorbye H, Sebjornsen S, et al. Clinical effect of temozolomide-based chemotherapy in poorly differentiated endocrine carcinoma after progression on first-line chemotherapy. Cancer 2011;**117**(20):4617-22.
9. Sorbye H, Welin S, Langer SW, et al. Predictive and prognostic factors for treatment and survival in 305 patients with advanced gastrointestinal neuroendocrine carcinoma (WHO G3): The NORDIC NEC study. Annals of Oncology 2013;**24**(1):152-60.
10. Hadoux J, Guigay J, Malka D, et al. Oxaliplatin-based chemotherapy for grade 3 neuroendocrine carcinoma after failure of platinum-based chemotherapy. European Neuro Endocrine Tumour Society 2013;**Abstract J2**.
11. Olsen IH, Knigge U, Federspiel B, et al. Topotecan Monotherapy in Heavily Pretreated Patients with Progressive Advanced Stage Neuroendocrine Carcinomas. Journal of Cancer 2014;**5**(8):628-32.
12. Heetfeld M, Chougnet CN, Olsen IH, et al. Characteristics and treatment of patients with G3 gastroenteropancreatic neuroendocrine neoplasms. Endocrine-Related Cancer 2015;**22**(4):657-64.
13. Olsen IH, Sørensen JB, Federspiel B, et al. Temozolomide as Second or Third Line Treatment of Patients with Neuroendocrine Carcinomas. The Scientific World Journal 2012;**2012**:1-4.
14. Yamaguchi T, Machida N, Morizane C, et al. Multicenter retrospective analysis of systemic chemotherapy for advanced neuroendocrine carcinoma of the digestive system. Cancer Sci 2014;**105**(9):1176-81.
15. McNamara MG, Frizziero M, Jacobs T, et al. Second-line treatment in patients (pts) with advanced extra-pulmonary poorly differentiated neuroendocrine carcinoma (EP-PD-NEC): A systematic review and meta-analysis. Neuroendocrinology 2019;**108**(Suppl 1):1-273.
16. Irinotecan hydrochloride Summary of Product Characteristics. https://www.medicines.org.uk/emc/product/5794/smpc#PHARMACOLOGICAL_PROPS (accessed 23 March 2016).
17. ONIVYDE 5 mg/ml concentrate for solution for infusion Summary of Product Characteristics. https://www.medicines.org.uk/emc/product/9200#PHARMACOLOGICAL_PROPS (accessed 01 July 2019).

For peer review only - http://bmjopen.bmj.com/site/about/guidelines.xhtml

18. Camptosar (irinotecan) U.S. Package Insert. http://www.accessdata.fda.gov/drugsatfda_docs/label/2014/020571s048lbl.pdf (accessed 23 March 2016).
19. Baker JH, Lam J, Kyle AH, et al. Irinophore C, a novel nanoformulation of irinotecan, alters tumor vascular function and enhances the distribution of 5-fluorouracil and doxorubicin. Clin Cancer Res 2008;**14**(22):7260-71.
20. Verreault M, Strutt D, Masin D, et al. Vascular normalization in orthotopic glioblastoma following intravenous treatment with lipid-based nanoparticulate formulations of irinotecan (Irinophore C), doxorubicin (Caelyx(R)) or vincristine. BMC Cancer 2011;**11**:124.
21. Chiang NJ, Chao TY, Hsieh RK, et al. A phase I dose-escalation study of PEP02 (irinotecan liposome injection) in combination with 5-fluorouracil and leucovorin in advanced solid tumors. BMC Cancer 2016;**16**(1):907.
22. Chen LT, Von Hoff DD, Li CP, et al. Expanded analyses of napoli-1: Phase 3 study of MM-398 (nal-IRI), with or without 5-fluorouracil and leucovorin, versus 5-fluorouracil and leucovorin, in metastatic pancreatic cancer (mPAC) previously treated with gemcitabine-based therapy. Journal of Clinical Oncology 2015;**33**(3_suppl):234-34.
23. NCCN guidelines: Small Cell Lung Cancer. https://www.nccn.org/professionals/physician_gls/pdf/sclc.pdf (accessed 05 Aug 2019).
24. NCCN guidelines: Non-Small Cell Lung Cancer. https://www.nccn.org/professionals/physician_gls/pdf/nscl.pdf (accessed 09 Sep 2019).
25. Fossella FV. Docetaxel for previously treated non-small-cell lung cancer. Oncology (Williston Park) 2002;**16**(6 Suppl 6):45-51.
26. Eisenhauer EA, Therasse P, Bogaerts J, et al. New response evaluation criteria in solid tumours: Revised RECIST guideline (version 1.1). European Journal of Cancer 2009;**45**(2):228-47.
27. Groenvold M, Klee MC, Sprangers MA, et al. Validation of the EORTC QLQ-C30 quality of life questionnaire through combined qualitative and quantitative assessment of patient-observer agreement. Journal of clinical epidemiology 1997;**50**(4):441-50.
28. Yadegarfar G, Friend L, Jones L, et al. Validation of the EORTC QLQ-GINET21 questionnaire for assessing quality of life of patients with gastrointestinal neuroendocrine tumours. British journal of cancer 2013;**108**(2):301-10.
29. Simon R, Wittes RE, Ellenberg SS. Randomized phase II clinical trials. Cancer Treat Rep 1985;**69**(12):1375-81.
30. A'Hern RP. Sample size tables for exact single-stage phase II designs. Stat Med 2001;**20**(6):859-66.
31. Consolidated Standards of Reporting Trials Guidelines. http://www.consort-statement.org/ (accessed 07 Dec 2018).
32. International Committee of Medical Journal Editors: Defining the Role of Authors and Contributors. http://www.icmje.org/recommendations/browse/roles-and-responsibilities/defining-the-role-of-authors-and-contributors.html (accessed 02 Jul 2019).
33. Information Commissioner's Office: Guide to the General Data Protection Regulation (GDPR). https://ico.org.uk/for-organisations/guide-to-data-protection/guide-to-the-general-data-protection-regulation-gdpr/ (accessed 02 Jul 2019).

For peer review only - http://bmjopen.bmj.com/site/about/guidelines.xhtml

SCREENING

Potentially eligible patients with progressive PD-EP-NEC (grade 3) will be identified at the standard clinic visit at which progression following first-line chemotherapy is discussed.

Written Informed Consent and REGISTRATION

Formal eligibility and pre-randomisation assessment (≤14 days prior to randomisation):

Full blood count, biochemistry, renal function, pregnancy test (if applicable) and electrocardiogram (ECG)

Quality of Life (QoL): EORTC QLQ-C30, EORTC QLQ-GI.NET21

RANDOMISATION (1:1)

Minimisation incorporating a random element, stratified by: hospital site, Ki-67 marker, ECOG performance status, presence of liver metastases and response to first-line platinum-based chemotherapy

Post-randomisation assessments: Blood samples and archival paraffin-embedded tissue for translational research.

Baseline CT scan ≤28 days prior to starting treatment.

Nal-IRI, 5-FU and folinic acid (n=51)

Nal-IRI (80mg/m^2 IV over 90 minutes) followed by folinic acid (as per local standard practice), followed by 5-FU (2400 mg /m^2 BSA intravenously over 46 hours) every 14 days (+3 days/-1 day) until progression, toxicity or ≥28 day delay

Full blood count, urea, electrolytes, and liver function tests ≤3 days of day 1 of each cycle

CT or MRI scan every 8 weeks (±7 days) until disease progression

QoL: day 1 of cycles 3, 5, 7, 9, etc. (every 6 weeks (±7 days) until disease progression)

Blood samples at start of treatment, 6 weekly thereafter and on progression

Docetaxel (n=51)

Docetaxel (75mg/m^2 IV over 60 minutes) every 21 days (+3 days/-1 day) until progression, toxicity or ≥28 day delay

Full blood count, urea, electrolytes, and liver function tests ≤3 days of day 1 of each cycle

CT or MRI scan every 8 weeks (±7 days) until disease progression

QoL: day 1 of cycles 2, 4, 6, 8, etc. (every 6 weeks (±7 days) until disease progression)

Blood samples at start of treatment, 6 weekly thereafter and on progression

Toxicity assessment: 28 days (+7 days) post end of treatment (telephone call or clinic visit as clinically appropriate)

For peer review only - http://bmjopen.bmj.com/site/about/guidelines.xhtml

Supplementary material

Contents

Supplementary material 1
1. TRIAL SITES 2
2. DOSE MODIFICATIONS 3
2.1 Liposomal irinotecan (nal-IRI) dose modifications........ 3
2.2 5-Fluorouracil (5-FU) and folinic acid dose modifications 5
2.2.1 Haematological toxicities: 5-FU dose modifications........ 5
2.2.2 Non-haematological toxicities: 5-FU dose modifications 5
2.2.3 Other toxicity requiring special attention........ 6
2.3 Docetaxel dose modifications........ 6
2.4 Management of infusion reactions........ 8

For peer review only

1. TRIAL SITES

Table 1 list the trial sites, which are taking part in the NET-02 trial, indicating if they are open to recruitment or are in set-up. The principal investigator (PI) of each site is listed and for sites that are open to recruitment, the number or patients randomised up to the point of submission are provided.

Table 1 | Trial sites

Site status	Site Name	PI Name	Number of patients randomised
Open	Christie Hospital	Dr Mairéad McNamara	8
	Weston Park Hospital	Dr Jon Wadsley	2
	Beatson	Professor Nick Reed	2
	Hammersmith Hospital	Dr Rohini Sharma	1
	Royal Free Hospital	Dr Daniel Krell	0
	Royal Marsden Hospital	Dr Ian Chau	1
	Western General Hospital	Dr Lucy Wall	1
	The Clatterbridge Cancer Centre	Dr Olusola Faluyi	1
	University Hospital Southampton	Dr Judith Cave	1
	Velindre Cancer Centre	Dr Carys Morgan	0
	Guy's Hospital	Dr Debashis Sarker	0
	Newcastle	Dr Jane Margetts	0
In set-up	University Hospital Coventry	Dr Sharmila Sothi	n/a
	Belfast	Dr Martin Eatock	n/a
	St James University Hospital	Dr Alan Anthoney	n/a
	Cheltenham General Hospital	Dr David Farrugia	n/a

2. DOSE MODIFICATIONS

2.1 Liposomal irinotecan (nal-IRI) dose modifications

For Grade 1 and 2 toxicities, no dose modifications are required. In the event of Grade 3 or 4 toxicity, the doses of liposomal irinotecan (nal-IRI) and 5-fluorouracil (5-FU) must be reduced and subsequent doses of nal-IRI and 5-FU must continue to be adjusted as indicated in Table 2. All dose modifications must be based on the worst preceding toxicity.

Table 2 | Dose modifications for nal-IRI and 5-FU and folinic acid, for Grade 3-4 toxicities.

	**Toxicity**<br>CTCAE Grade (value)	**Dose Adjustment**
**Haematological toxicities**	**Neutropenia**<br>Grade 3 or Grade 4<br>(<1000/mm^3: <1x10^9/L)<br>Or neutropenic fever	**A new cycle of therapy should not begin until the absolute neutrophil count is ≥1.5x10^9/L (dose modifications below are for subsequent treatments, if grade 3 or 4 neutropenia is recorded on day 1 of a cycle or neutropenic fever is experienced during a cycle)***<br>**First occurrence** Reduce nal-IRI dose to 60 mg/m^2<br>Reduce 5-FU dose by 25% (1800 mg/m^2)<br>**Second occurrence** Reduce nal-IRI dose to 50 mg/m^2<br>Reduce 5-FU dose by 25% (1350 mg/m^2)<br>**Third occurrence** Discontinue treatment
	**Thrombocytopenia**<br>**Leukopenia**<br>Grade 3 or 4	**A new cycle of therapy should not begin until the platelet count is ≥100x10^9/L**<br>Dose modifications for grade 3 or 4 thrombocytopenia are the same as recommended for neutropenia above for first, second and third recurrence.
**Non-haematological toxicities**	**Diarrhoea**<br>Grade 3 or 4<br>(≥7 stools per day pre-treatment)	**A new cycle of therapy should not begin until diarrhoea resolves to ≤Grade 1** (2-3 stools/day more than pre-treatment frequency) **(Dose modifications below are for subsequent treatments)**<br>**First occurrence** Reduce nal-IRI dose to 60 mg/m^2<br>Reduce 5-FU dose by 25% (1800 mg/m^2)<br>**Second occurrence** Reduce nal-IRI dose to 50 mg/m^2<br>Reduce 5-FU dose by 25% (1350 mg/m^2)

For peer review only

	Toxicity<br>CTCAE Grade (value)	Dose Adjustment
		**Third occurrence** Discontinue treatment
	**Nausea/vomiting**<br>Grade 3 or 4 despite optimal antiemetic therapy	**A new cycle of therapy should not begin until nausea/vomiting resolves to ≤Grade 1 or baseline (Dose modifications below are for subsequent treatments)**<br>**First occurrence** Optimise antiemetic therapy<br>Reduce nal-IRI dose to 60 mg/m^2<br>**Second occurrence** Optimise antiemetic therapy<br>Reduce nal-IRI dose to 50 mg/m^2<br>**Third occurrence** Discontinue treatment
	**Hepatic, renal, respiratory or other toxicities**<br>Grade 3 or 4<br>(asthenia and grade 3 anorexia do not require dose adjustment and also excluding grade ≥3 ALT/AST which resolve to baseline within 7 days and grade ≥3 toxicities which following case causality assessment are not in the category of 'Certain', 'Probable' or 'Possible' and as such are not related to study treatment, or which are not considered a clinically-significant toxicity)	**A new cycle of therapy should not begin until the adverse reaction resolves to ≤Grade 1 (Dose modifications below are for subsequent treatments)**<br>**First occurrence** Reduce nal-IRI dose to 60 mg/m^2<br>Reduce 5-FU dose by 25% (1800 mg/m^2)<br>**Second occurrence** Reduce nal-IRI dose to 50 mg/m^2<br>Reduce 5-FU dose by 25% (1350 mg/m^2)<br>**Third occurrence** Discontinue treatment

* Prophylactic use of granulocyte colony-stimulating factor (G-CSF) can be considered prior to dose modification in those patients who have had at least one episode of grade 3 or 4 neutropenia or neutropenic fever while receiving therapy or have had documented grade 3 or 4 neutropenia or neutropenic fever while receiving prior anti-neoplastic therapy.
CTCAE: Common Terminology Criteria for Adverse Events, ALT: alanine aminotransferase, AST: aspartate aminotransferase.

For peer review only

2.2 5-Fluorouracil (5-FU) and folinic acid dose modifications

Dose modifications for 5-FU are provided below. No dose adjustments for toxicity are required for folinic acid. Folinic acid must be given immediately prior to each 5-FU dose; hence, if the 5-FU dose is held, folinic acid dose should be held as well. If the dosing of nal-IRI needs to be withheld, then the 5-FU/folinic acid in the combination can be administered as monotherapy. In case a patient experiences an infusion reaction, either institutional guidelines or the guidelines provided for nal-IRI infusion reaction management (section 2.4) should be used.

2.2.1 Haematological toxicities: 5-FU dose modifications

Absolute neutrophil count (ANC) and platelet count should be measured locally no more than 3 days prior to day 1 of each treatment cycle. Treatment should only proceed if;

- ANC $\geq 1.5\text{x}10^9$/L
- Platelet count $\geq 100\text{x}10^9$/L

In the event of haematological toxicity, treatment should be delayed (up to 28 days) to allow sufficient time for recovery. On recovery, treatment should be administered according to the guidelines provided in Table 2.

2.2.2 Non-haematological toxicities: 5-FU dose modifications

Treatment should be delayed until all clinically significant Grade 3 or 4 non-haematological toxicities resolve to Grade 1 or baseline. If delays are greater than 28 days for toxicity, the participant should be withdrawn from trial treatment. Dose adjustments of other 5-FU-related toxicities are provided in Table 3. Asthenia, grade 3 anorexia and grade ≥3 toxicities which following case causality assessment are not in the category of ‘Certain’, ‘Probable’ or ‘Possible’ and as such are not related to study treatment, or which are not considered a clinically-significant toxicity, do not require dose modifications.

For peer review only

Table 3 | 5-FU dose modifications for other non-haematological toxicities

Worst toxicity CTCAE grade	5-FU dose for next cycle[a]
Grade 1 or 2	100% of previous dose, except for Grade 2 hand-foot syndrome, Grade 2 cardiac toxicity, or any grade neurocerebellar toxicity
Grade 2 hand-foot syndrome	Reduce dose by 25%[b]
Any grade neurocerebellar or ≥ Grade 2 cardiac toxicity	Discontinue therapy
Grade 3 or 4	Reduce dose by 25%[b], except for Grade 3 or 4 hand-foot syndrome
Grade 4 or 4 hand-foot syndrome	Discontinue therapy

[a] All dose modifications must be based on the worst preceding toxicity.

[b] Participants who require more than 2 dose reductions should be withdrawn from trial treatment unless agreed with the Chief Investigator or delegate.

CTCAE: Common Terminology Criteria for Adverse Events, 5-FU; 5-Fluorouracil.

2.2.3 Other toxicity requiring special attention

Corrected QT interval (QTc) prolongation that occurs in the setting of diarrhoea-induced electrolyte imbalance should be treated with appropriate electrolyte repletion. Once the underlying abnormality is corrected and the electrocardiogram (ECG) abnormalities have reversed, treatment may continue under careful monitoring, and with appropriate dose modification for diarrhoea as per local standard of care practice.

2.3 Docetaxel dose modifications

Neutrophil and platelet count should be measured locally no more than 3 days prior to day 1 of each treatment cycle. Treatment should only proceed if:

- ANC ≥ $1.5x10^9/L$
- Platelet count ≥$100x10^9/L$
- Bilirubin ≤1.5 x upper limit of normal (ULN)
- Alanine aminotransferase (ALT) and/or Aspartate aminotransferase (AST) ≤2.5 x ULN (in absence of liver metastasis) or ≤5 x ULN (in presence of liver metastasis)

In the event of haematological toxicity, treatment should be delayed (up to 28 days) to allow sufficient time for recovery. Guidelines for docetaxel dose modifications are provided in Table 4.

For peer review only

Table 4 | Dose reductions for docetaxel toxicities

Toxicity	Severity	Management
**Hypersensitivity**	Grade 3/Grade 4	Administration of appropriate medication (see below).
**Neutropenia**	Day 1 neutrophil count <1500/mm^3: <1.5x10^9/L	Stop treatment until neutrophils recovers to at least 1.5x10^9/L. If neutrophils <1.5x10^9/L for ≤7 days, restart docetaxel at full dose (75mg/m^2). If neutrophils <1.5x10^9/L for >7 days, restart docetaxel at 55mg/m^2 or next lowest dose level (40mg/m^2) if already reduced.*
	Febrile neutropenia OR prolonged Grade 4 neutropenia (Neutrophil count <500/mm^3: <0.5x10^9/L for 7 days or more)	Stop treatment until neutrophils ≥ 1.5x10^9/L. Restart drug at 55mg/m^2 or next lowest dose level (40mg/m^2) if already reduced.*
**Neuropathy**	Grade 3/Grade 4	Stop docetaxel treatment.
**Thrombocytopenia**	Platelet count <100 x 10^9/L	Stop treatment until platelets ≥100x^9/L. Restart drug at full dose (75mg/m^2).
	Platelet count <50 x 10^9/L (Grade 3/Grade 4)	Stop treatment until platelets ≥100x^9/L. Restart drug at 55mg/m^2 or next lowest dose level (40mg/m^2) if already reduced.
**Hepatic Dysfunction**	Bilirubin >1.5 ULN<br>ALT/AST>2.5 x ULN (in absence of liver metastasis), >5 x ULN (in presence of liver metastasis)	Stop docetaxel until parameters recover to baseline levels. Restart drug at 55mg/m^2 or next lowest dose level (40mg/m^2) if already reduced.
**Cutaneous reaction**	Grade 2	Stop treatment until recovery to Grade 1 or better. Restart drug at full dose (75mg/m^2).
	Severe or cumulative<br>(Grade 3/Grade 4)	Stop treatment until recovery (Grade 1 or better). Restart drug at 55 mg/m^2 or next

For peer review only

Toxicity	Severity	Management
		lowest dose level (40mg/m2) if already reduced.
**Other non-haematological toxicity (excluding grade ≥3 toxicities which following case causality assessment are not in the category of 'Certain', 'Probable' or 'Possible' and as such is not related to study treatment, or which are not considered a clinically-significant toxicity)**	Grade 3/Grade 4	Stop docetaxel until parameters recover to baseline levels. Restart drug at 55mg/m^2 or next lowest dose level (40mg/m^2) if already reduced.

* Prophylactic use of granulocyte colony-stimulating factor (G-CSF) can be considered prior to dose modification in those patients who have had at least one episode of grade 3 or 4 neutropenia or neutropenic fever while receiving therapy or have had documented grade 3 or 4 neutropenia or neutropenic fever while receiving prior anti-neoplastic therapy.
ULN: upper limit of normal, ALT: alanine aminotransferase, AST: aspartate aminotransferase.

2.4 Management of infusion reactions

The guidelines described in this section can be followed in case of infusion reactions. Infusion reactions will be defined according to the National Cancer Institute Common Terminology Criteria for Adverse Events (CTCAE) (Version 5.0) definitions of an allergic reaction or anaphylaxis. Institutional policies or the following treatment guidelines shall be used for the management of infusion reactions.

Grade 1

- Slow infusion rate by 50%.
- Monitor patient every 15 minutes for worsening of condition.
- Future infusions may be administered at a reduced rate (e.g. over 120 minutes for nal-IRI), at the discretion of the Investigator.

Grade 2

- Stop infusion.

For peer review only

- Administer diphenhydramine hydrochloride 50 mg intravenously (IV) (or similar), acetaminophen 650 mg (or similar) orally, and oxygen.
- Resume infusion at 50% of the prior rate once infusion reaction has resolved.
- Monitor patient every 15 minutes for worsening of condition.
- For all subsequent infusions, pre-medicate with diphenhydramine hydrochloride 50 mg IV (or similar), dexamethasone 10 mg IV, and acetaminophen 650 mg (or similar) orally.
- Future infusions may be administered at a reduced rate (e.g. over 120 minutes for nal-IRI), at the discretion of the Investigator.

Grade 3

- Stop infusion and disconnect infusion tubing from patient.
- Administer diphenhydramine hydrochloride 50 mg IV (or similar), dexamethasone 10 mg IV, bronchodilators for bronchospasm, and other medications or oxygen as medically necessary.
- No further treatment will be permitted.

Grade 4

- Stop the infusion and disconnect infusion tubing from patient.
- Administer epinephrine (adrenaline), bronchodilators or oxygen as indicated for bronchospasm.
- Administer diphenhydramine hydrochloride 50 mg IV (or similar), dexamethasone 10 mg IV and other medications as medically necessary.
- Consider hospital admission for observation.
- No further treatment will be permitted.

For peer review only

For patients who experience a Grade 1 or Grade 2 infusion reaction, future infusions may be administered at a reduced rate (over 120 minutes), at the discretion of the treating physician. For patients who experience a second grade 1 or 2 infusion reaction, administer dexamethasone 10 mg IV. All subsequent infusions should be pre-medicated with diphenhydramine hydrochloride 50 mg IV (or similar), dexamethasone 10 mg IV, and acetaminophen 650 mg orally (or similar).

For peer review only

Reporting checklist for protocol of a clinical trial.

Based on the SPIRIT guidelines.

Instructions to authors

Complete this checklist by entering the page numbers from your manuscript where readers will find each of the items listed below.

Your article may not currently address all the items on the checklist. Please modify your text to include the missing information. If you are certain that an item does not apply, please write "n/a" and provide a short explanation.

Upload your completed checklist as an extra file when you submit to a journal.

In your methods section, say that you used the SPIRITreporting guidelines, and cite them as:

Chan A-W, Tetzlaff JM, Altman DG, Laupacis A, Gøtzsche PC, Krleža-Jerić K, Hróbjartsson A, Mann H, Dickersin K, Berlin J, Doré C, Parulekar W, Summerskill W, Groves T, Schulz K, Sox H, Rockhold FW, Rennie D, Moher D. SPIRIT 2013 Statement: Defining standard protocol items for clinical trials. Ann Intern Med. 2013;158(3):200-207

		Reporting Item	Page Number
**Administrative information**			
Title	#1	Descriptive title identifying the study design, population, interventions, and, if applicable, trial acronym	1

For peer review only

Trial registration	#2a	Trial identifier and registry name. If not yet registered, name of intended registry	2, 19
Trial registration: data set	#2b	All items from the World Health Organization Trial Registration Data Set	Can be accessed via ISRCTN website using the ISRCTN on page 2
Protocol version	#3	Date and version identifier	19
Funding	#4	Sources and types of financial, material, and other support	19, 20
Roles and responsibilities: contributorship	#5a	Names, affiliations, and roles of protocol contributors	1, 20
Roles and responsibilities: sponsor contact information	#5b	Name and contact information for the trial sponsor	20
Roles and responsibilities: sponsor and funder	#5c	Role of study sponsor and funders, if any, in study design; collection, management, analysis, and interpretation of data; writing of the report; and the decision to submit the report for publication, including whether they will have ultimate authority over any of these activities	19

For peer review only

Roles and responsibilities: committees	#5d	Composition, roles, and responsibilities of the coordinating centre, steering committee, endpoint adjudication committee, data management team, and other individuals or groups overseeing the trial, if applicable (see Item 21a for data monitoring committee)	18
**Introduction**			4-7
Background and rationale	#6a	Description of research question and justification for undertaking the trial, including summary of relevant studies (published and unpublished) examining benefits and harms for each intervention	
Background and rationale: choice of comparators	#6b	Explanation for choice of comparators	5-6
Objectives	#7	Specific objectives or hypotheses	7-8
Trial design	#8	Description of trial design including type of trial (eg, parallel group, crossover, factorial, single group), allocation ratio, and framework (eg, superiority, equivalence, non-inferiority, exploratory)	8-9
**Methods: Participants,**			

For peer review only

**interventions, and outcomes**			
Study setting	#9	Description of study settings (eg, community clinic, academic hospital) and list of countries where data will be collected. Reference to where list of study sites can be obtained	12
Eligibility criteria	#10	Inclusion and exclusion criteria for participants. If applicable, eligibility criteria for study centres and individuals who will perform the interventions (eg, surgeons, psychotherapists)	9-11
Interventions: description	#11a	Interventions for each group with sufficient detail to allow replication, including how and when they will be administered	13-14
Interventions: modifications	#11b	Criteria for discontinuing or modifying allocated interventions for a given trial participant (eg, drug dose change in response to harms, participant request, or improving / worsening disease)	14
Interventions: adherance	#11c	Strategies to improve adherence to intervention protocols, and any procedures for monitoring adherence (eg, drug tablet return; laboratory tests)	N/A – treatment administered in hospital
Interventions: concomitant care	#11d	Relevant concomitant care and interventions that are permitted or prohibited during the trial	14

For peer review only

Outcomes	#12	Primary, secondary, and other outcomes, including the specific measurement variable (eg, systolic blood pressure), analysis metric (eg, change from baseline, final value, time to event), method of aggregation (eg, median, proportion), and time point for each outcome. Explanation of the clinical relevance of chosen efficacy and harm outcomes is strongly recommended	7-8 & 16-17
Participant timeline	#13	Time schedule of enrolment, interventions (including any run-ins and washouts), assessments, and visits for participants. A schematic diagram is highly recommended (see Figure)	12-15 & Figure 1
Sample size	#14	Estimated number of participants needed to achieve study objectives and how it was determined, including clinical and statistical assumptions supporting any sample size calculations	11
Recruitment	#15	Strategies for achieving adequate participant enrolment to reach target sample size	12

Methods:

Assignment of

For peer review only

**interventions (for controlled trials)**			
Allocation: sequence generation	#16a	Method of generating the allocation sequence (eg, computer-generated random numbers), and list of any factors for stratification. To reduce predictability of a random sequence, details of any planned restriction (eg, blocking) should be provided in a separate document that is unavailable to those who enrol participants or assign interventions	13
Allocation concealment mechanism	#16b	Mechanism of implementing the allocation sequence (eg, central telephone; sequentially numbered, opaque, sealed envelopes), describing any steps to conceal the sequence until interventions are assigned	13
Allocation: implementation	#16c	Who will generate the allocation sequence, who will enrol participants, and who will assign participants to interventions	12-13
Blinding (masking)	#17a	Who will be blinded after assignment to interventions (eg, trial participants, care providers, outcome assessors, data analysts), and how	N/A – Open label trial

For peer review only

Blinding (masking): emergency unblinding	#17b	If blinded, circumstances under which unblinding is permissible, and procedure for revealing a participant's allocated intervention during the trial	N/A – Open label trial
**Methods: Data collection, management, and analysis**			
Data collection plan	#18a	Plans for assessment and collection of outcome, baseline, and other trial data, including any related processes to promote data quality (eg, duplicate measurements, training of assessors) and a description of study instruments (eg, questionnaires, laboratory tests) along with their reliability and validity, if known. Reference to where data collection forms can be found, if not in the protocol	12-13 & 15-16
Data collection plan: retention	#18b	Plans to promote participant retention and complete follow-up, including list of any outcome data to be collected for participants who discontinue or deviate from intervention protocols	14

For peer review only

Data management	#19	Plans for data entry, coding, security, and storage, including any related processes to promote data quality (eg, double data entry; range checks for data values). Reference to where details of data management procedures can be found, if not in the protocol	15-16
Statistics: outcomes	#20a	Statistical methods for analysing primary and secondary outcomes. Reference to where other details of the statistical analysis plan can be found, if not in the protocol	16-17
Statistics: additional analyses	#20b	Methods for any additional analyses (eg, subgroup and adjusted analyses)	17
Statistics: analysis population and missing data	#20c	Definition of analysis population relating to protocol non-adherence (eg, as randomised analysis), and any statistical methods to handle missing data (eg, multiple imputation)	16
**Methods: Monitoring**			
Data monitoring: formal committee	#21a	Composition of data monitoring committee (DMC); summary of its role and reporting structure; statement of whether it is independent from the sponsor and competing interests; and reference to where further details about its charter can be found, if not in the	18

For peer review only

		protocol. Alternatively, an explanation of why a DMC is not needed	
Data monitoring: interim analysis	#21b	Description of any interim analyses and stopping guidelines, including who will have access to these interim results and make the final decision to terminate the trial	16
Harms	#22	Plans for collecting, assessing, reporting, and managing solicited and spontaneously reported adverse events and other unintended effects of trial interventions or trial conduct	15
Auditing	#23	Frequency and procedures for auditing trial conduct, if any, and whether the process will be independent from investigators and the sponsor	18
**Ethics and dissemination**			
Research ethics approval	#24	Plans for seeking research ethics committee / institutional review board (REC / IRB) approval	19
Protocol amendments	#25	Plans for communicating important protocol modifications (eg, changes to eligibility criteria, outcomes, analyses) to relevant parties (eg, investigators, REC / IRBs, trial participants, trial registries, journals, regulators)	18

For peer review only

Consent or assent	#26a	Who will obtain informed consent or assent from potential trial participants or authorised surrogates, and how (see Item 32)	12
Consent or assent: ancillary studies	#26b	Additional consent provisions for collection and use of participant data and biological specimens in ancillary studies, if applicable	13
Confidentiality	#27	How personal information about potential and enrolled participants will be collected, shared, and maintained in order to protect confidentiality before, during, and after the trial	19
Declaration of interests	#28	Financial and other competing interests for principal investigators for the overall trial and each study site	20-21
Data access	#29	Statement of who will have access to the final trial dataset, and disclosure of contractual agreements that limit such access for investigators	19
Ancillary and post trial care	#30	Provisions, if any, for ancillary and post-trial care, and for compensation to those who suffer harm from trial participation	N/A – no special compensation arrangements beyond rights as an NHS patient.

For peer review only

Dissemination policy: trial results	#31a	Plans for investigators and sponsor to communicate trial results to participants, healthcare professionals, the public, and other relevant groups (eg, via publication, reporting in results databases, or other data sharing arrangements), including any publication restrictions	19
Dissemination policy: authorship	#31b	Authorship eligibility guidelines and any intended use of professional writers	19
Dissemination policy: reproducible research	#31c	Plans, if any, for granting public access to the full protocol, participant-level dataset, and statistical code	N/A – unknown at this stage
**Appendices**			
Informed consent materials	#32	Model consent form and other related documentation given to participants and authorised surrogates	Not submitted
Biological specimens	#33	Plans for collection, laboratory evaluation, and storage of biological specimens for genetic or molecular analysis in the current trial and for future use in ancillary studies, if applicable	13, 15

None The SPIRIT checklist is distributed under the terms of the Creative Commons Attribution License CC-BY-ND 3.0. This checklist can be completed online using https://www.goodreports.org/, a tool made by the EQUATOR Network in collaboration with Penelope.ai
